# Unsupervised Detection of Cell-Assembly Sequences by Similarity-Based Clustering

**DOI:** 10.3389/fninf.2019.00039

**Published:** 2019-05-31

**Authors:** Keita Watanabe, Tatsuya Haga, Masami Tatsuno, David R. Euston, Tomoki Fukai

**Affiliations:** ^1^Department of Complexity Science and Engineering, University of Tokyo, Kashiwa, Japan; ^2^RIKEN Center for Brain Science, Wako, Japan; ^3^Department of Neuroscience, Canadian Center for Behavioral Neuroscience, University of Lethbridge, Lethbridge, AB, Canada; ^4^Neural Coding and Brain Computing Unit, Okinawa Institute of Science and Technology, Okinawa, Japan

**Keywords:** neural ensemble, neural code, behavioral information, multi-neuron recordings, data mining, place cells, prefrontal neurons

## Abstract

Neurons which fire in a fixed temporal pattern (i.e., “cell assemblies”) are hypothesized to be a fundamental unit of neural information processing. Several methods are available for the detection of cell assemblies without a time structure. However, the systematic detection of cell assemblies with time structure has been challenging, especially in large datasets, due to the lack of efficient methods for handling the time structure. Here, we show a method to detect a variety of cell-assembly activity patterns, recurring in noisy neural population activities at multiple timescales. The key innovation is the use of a computer science method to comparing strings (“edit similarity”), to group spikes into assemblies. We validated the method using artificial data and experimental data, which were previously recorded from the hippocampus of male Long-Evans rats and the prefrontal cortex of male Brown Norway/Fisher hybrid rats. From the hippocampus, we could simultaneously extract place-cell sequences occurring on different timescales during navigation and awake replay. From the prefrontal cortex, we could discover multiple spike sequences of neurons encoding different segments of a goal-directed task. Unlike conventional event-driven statistical approaches, our method detects cell assemblies without creating event-locked averages. Thus, the method offers a novel analytical tool for deciphering the neural code during arbitrary behavioral and mental processes.

## 1. Introduction

Uncovering neural codes is of fundamental importance in neuroscience. Several experimental results suggest that synchronous or sequential firing of cortical neurons play active roles in primates (Abeles et al., 1993; Hatsopoulos et al., 1998; Steinmetz et al., 2000). In the rat somatosensory and auditory cortices, spontaneous and stimulus-evoked activities exhibit repeating sequences of neuronal firing (Luczak et al., 2007, 2009). In a rodent hippocampus, place cells exhibit precisely timed, repeating firing sequences representing the rat's trajectory, subsections of which repeat during each theta cycle (O'Keefe, 1976; Mehta et al., 2002; Villette et al., 2015). These sequences are replayed at compressed temporal scales during awake immobile and sleep states (Lee and Wilson, 2002; Foster and Wilson, 2006; Carr et al., 2011; Buzsáki and Moser, 2013) presumably for memory consolidation (Girardeau et al., 2009; Jadhav et al., 2012). Similar replay events have also been observed in the rodent prefrontal cortex (Euston et al., 2007).

The rapid development of techniques for large-scale recordings of neuronal activity provide fertile ground for the analysis of spike sequences. Calcium imaging enables simultaneous measurement of spike rates from hundreds to thousands of neurons (Kerr et al., 2005; Sasaki et al., 2007; Vogelstein et al., 2010; Deneux et al., 2016; Pnevmatikakis et al., 2016), and imaging by voltage indicators may further overcome the poor temporal resolution in imaging (Emiliani et al., 2015; Grinvald and Petersen, 2015; Knöpfel et al., 2015). Extracellular recording of neural activity with multi-electrodes has also evolved, allowing access to spike trains from large numbers of neurons (Buzsáki, 2004; Einevoll et al., 2012; Buzsáki et al., 2015).

Despite this progress in experimental techniques, methods for analyzing the spatiotemporal structure of cell assemblies are still limited (Chen and Wilson, 2017). Template matching is a standard technique for the detection of repeated activity patterns (Abeles et al., 1993; Kerr et al., 2005; Tatsuno et al., 2006; Euston et al., 2007; Luczak et al., 2007, 2009; Sasaki et al., 2008; Vogelstein et al., 2010). However, the method requires reference events, such as sensory cues and motor responses, and is easily disrupted by biological noise, such as jitters in spike timing and variations in sequence length. On the other hand, only a few studies have attempted the blind detection of cell-assembly sequences without relying on reference events (Shimazaki et al., 2012; Picado-Muiño et al., 2013; Torre et al., 2016; Quaglio et al., 2017; Russo et al., 2017), and such data analysis remains a challenge.

Here, we develop a method to detect self-similar firing patterns within cell assemblies using the edit similarity score. Edit similarity is a metric originally introduced in computer science to measure the distance between arbitrary strings and has been utilized for analyzing various types of sequences in computer science and biology (Navarro, 2001). Edit similarity measures matching between two sequences with flexible temporal alignment, which is an essential feature for detecting noisy spatiotemporal patterns embedded in neural activity. We extend the edit similarity score to a form applicable to neural activity data and develop a clustering method for blind cell-assembly detection. We evaluated the performance of the method with artificial data and found that our method is more robust against background noise than conventional clustering methods. Furthermore, we applied our method to experimental data recorded from the rat hippocampus (Mizuseki et al., 2009) and prefrontal cortex (Euston et al., 2007), and the algorithm detected several multi-cell sequences linked with behavior in an unsupervised manner. Robustness to noise and computational efficiency of our method will help the exhaustive search of repeated spatiotemporal patterns in large-scale neural data, which may lead to the elucidation of hidden neural codes.

## 2. Materials and Methods

We explain three major steps of the proposed method, i.e., edit similarity score with an exponentially growing gap penalty, clustering algorithms and profile generation algorithm.

### 2.1. Edit Similarity Calculation by N-W Algorithm

We explain the fundamentals of edit similarity score without gap penalty since this metric is not commonly used in neuroscience. Edit similarity score, or edit distance, quantifies the similarity between two strings with the minimum number of operations required to transform one string into the other. We can define arbitrary scoring schemes for each manipulation on strings, that is, insertion of a gap, deletion of a character, and comparison of two characters for coincidence. Needleman and Wunsch (1970) proposed one of the most widely used evaluation algorithms of this metric (N-W algorithm).

**Figure 1:**
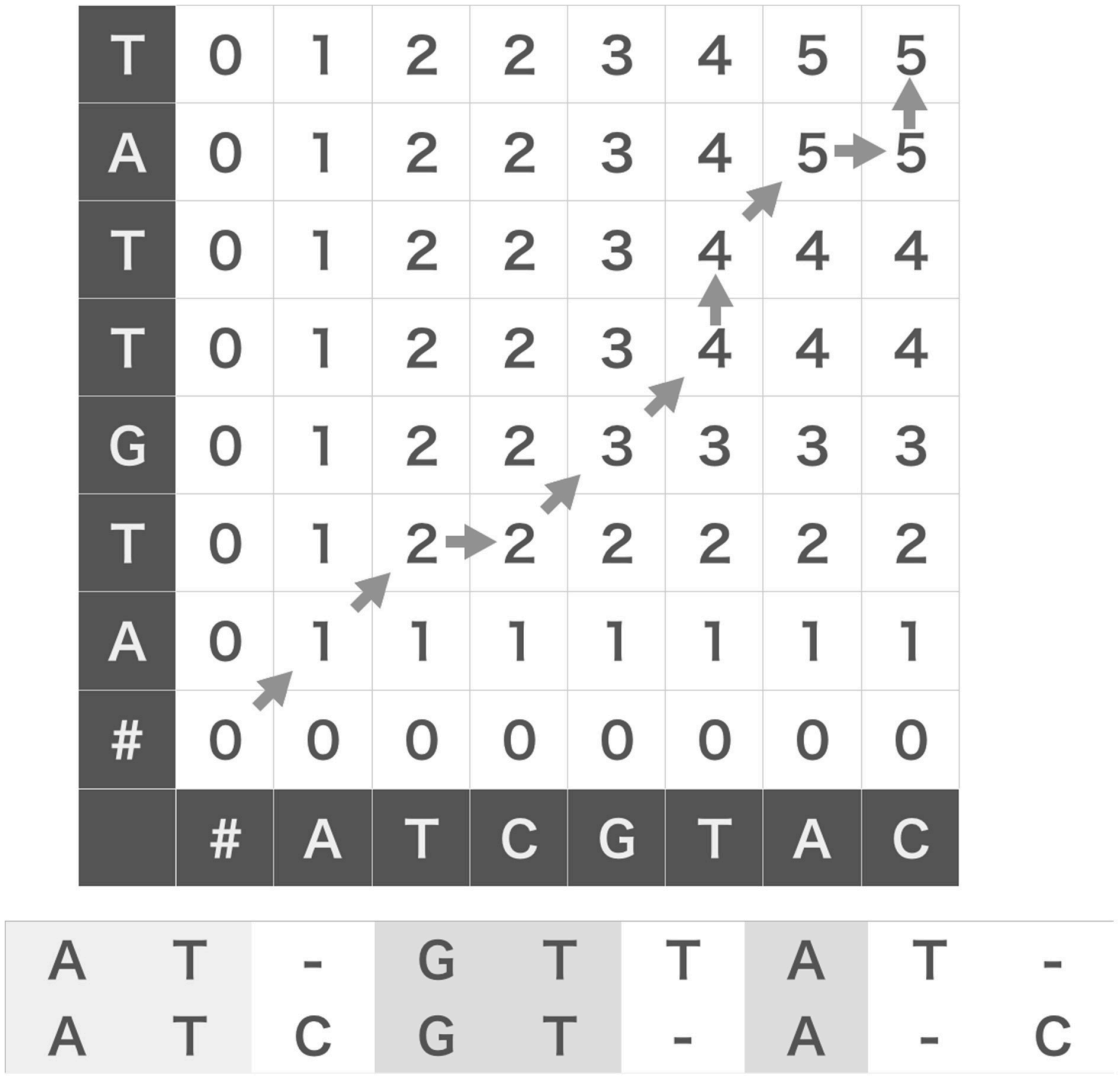
Edit similarity calculation between strings. We can calculate edit similarity score between “ATCGTAC” and “ATGTTAT” by using a dynamic programing table (top). Denoting the characters appearing after diagonal-up moves in the table gives a maximally coincident string (bottom).

The original N-W algorithm uses Dynamic Programming (DP) algorithm, which essentially partitions given problem into subsequent small subproblems to compose a solution of the original problem from those of the subproblems. As an example, we evaluate the score between two strings, W(1) = ATCGTAC and W(2) = ATGTTAT. As shown in Figure 1, we prepare a grid (DP table) and arrange the two strings along the abscissa and ordinate of the DP table. We add a null character “#” to the heads of the two strings and fill the leftmost column and the bottom row with zeros to initialize the following iterative operation.

We assign an appropriate score to each operation (insertion, deletion and coincidence). For the sake of simplicity, in this example without gap penalty we assign +1 to a coincidence, 0 to an insertion and a deletion. Let $\epsilon_{j}^{i}$ be the number of partial coincidences obtained up to the *i-*th element of *W*(1) and the *j-*th element of *W*(2). Then, we determine the value $\epsilon_{j}^{i}$ of the cell (*i, j*) of DP table using the following recursive equation:

(1) $$\begin{array}{l} \epsilon_{j}^{i}=\max\{\begin{array}{c} \epsilon_{j}^{i-1}\\ \epsilon_{j-1}^{i}\\ \epsilon_{j-1}^{i-1}+\delta \left(W\left(1\right)\left[j\right],W\left(2\right)\left[i\right]\right) \end{array}, \end{array}$$

where δ(*x, y*) is the Kronecker's delta: δ(*x, y*) = 1 if *x* = *y* and 0 if *x* ≠ *y*. We can fill the grid from the lower left cell to the top right cell with the scores calculated by the above equation (Figure 1). We note that δ(*x, #*) = 0 for any character *x* including a null character itself. Then, we obtain the similarity score of two strings **W**(1) and **W**(2), which is five in this case, at the top right cell $\epsilon_{8}^{8}$. Note that the operation $\epsilon_{j}^{i}=\epsilon_{j}^{i-1}$ corresponds to a deletion of *W*(2)[*i*], or equivalently, a gap insertion after *W*(1)[*j*]. Likewise, $\epsilon_{j}^{i}=\epsilon_{j-1}^{i}$ corresponds to a deletion of *W*(1)[*j*] or a gap insertion after *W*(2)[*i*], and $\epsilon_{j}^{i}=\epsilon_{j-1}^{i-1}+1$ corresponds to taking a coincidence.

The DP table enables us to obtain the substring that provides the maximum number of coincidences. For this purpose, we usually use the “β table” that stores the procedural dependency among the cells in the DP table (Needleman and Wunsch, 1970): from the top to the bottom of the rules shown above, we assign “start,” “upward” (↑), “rightward” (→), and “diagonal up” (↗) to the corresponding element of the β table. We can obtain a maximally coincident substring (ATGTA in the example) by back-tracking allowing in the β table from the top right cell to the left bottom cell and aligning the characters that appear after every “diagonal up” move. This procedure is illustrated with gray arrows in Figure 1, and the resultant alignments of *W*(1) and *W*(2) are shown at the bottom.

### 2.2. Extended N-W Algorithm for Neural Activity

**Figure 2:**
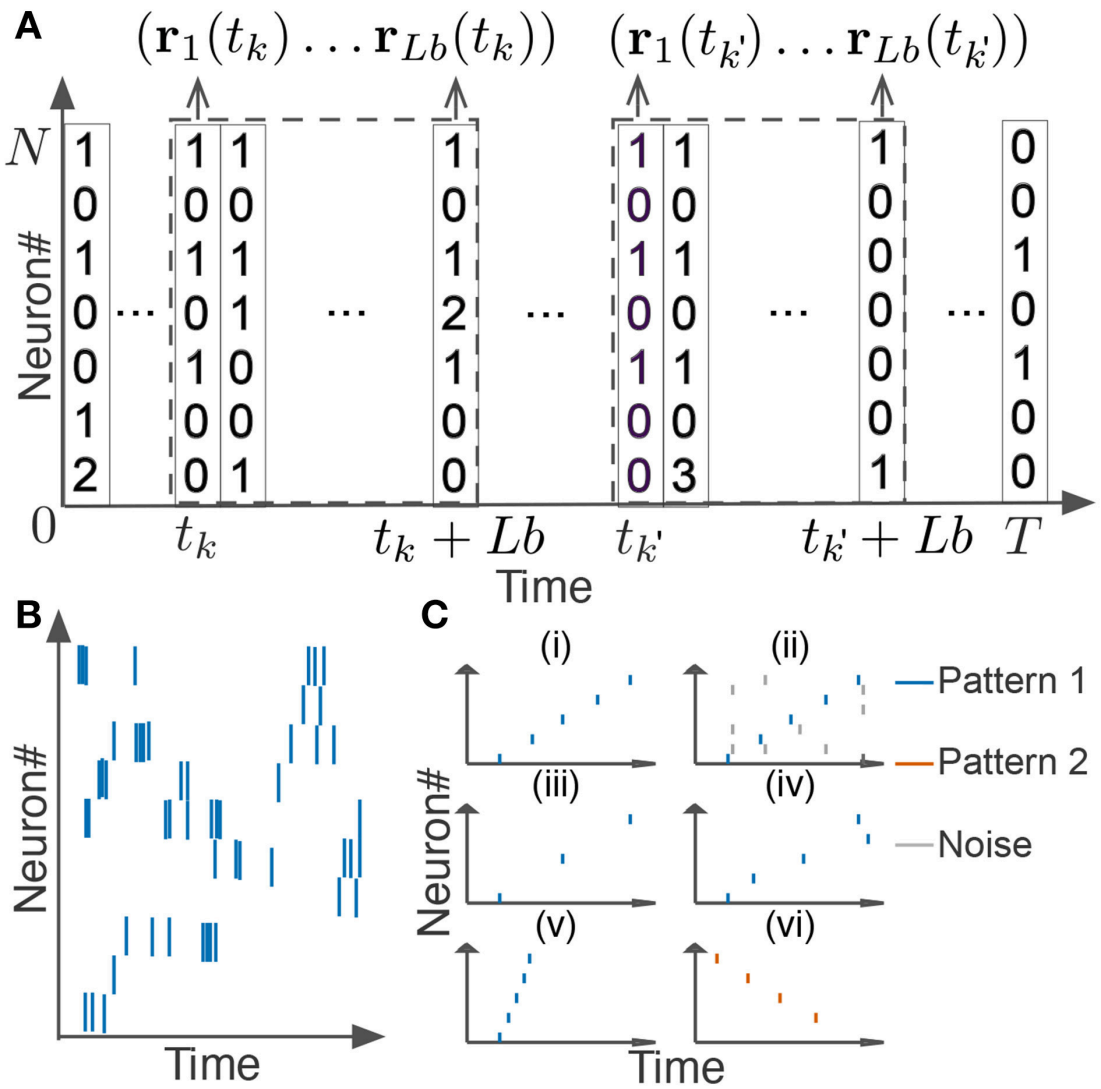
Detection of repetitive cell-assembly sequences. **(A)** Sliding time windows *W*(*t*_*k*_) are divided into *L* bins with an identical size, where *t*_*k*_ refers to the start time of the *k*-th time window. Population rate vector consists of the spike counts of individual neurons in each bin. **(B)** A temporal pattern in a sliding time window is schematically illustrated. Such a pattern may contain neurons belonging to a cell assembly as well as non-member neurons. Member neurons may fire at different rates with different temporal precision. Similarity between cell assembly sequences will increase when they share more member neurons and when they fire in a more similar temporal order at similar firing rates with higher temporal precision. Note that each member neuron may appear multiple times at different temporal positions in a time window. **(C)** Difficulties in detecting repetitive cell-assembly sequences are schematically illustrated. Blue and red bars show spikes of member neurons, while gray bars represent noisy spikes of non-member neurons: **(i)** temporal structure of target sequence; **(ii)** contamination by spikes of non-member neurons; **(iii)** missing spikes of member neurons; **(iv)** jitters in spike timing of member neurons; **(v)** arbitrary scaling of sequence length; **(vi)** member overlaps between different sequences.

In this section, we explain how we extended the original scoring method and algorithm for neural activity. We segmented spike data with a sliding time window of width *T*_*w*_, and divided each time window into *L* bins with size *b* (thus, *L* = *T*_*w*_/*b*). If we consider the activity pattern of the neural ensemble in each bin (i.e., the rate vector **r** of coincidently firing neurons in Figure 2A) as a “letter,” we obtain a string of letters in each time window. Note that each neuron may fire multiple spikes in a bin, so each component of the activity vector represents the number of spikes generated by the corresponding neuron in the bin. The window size and bin size are manually determined from the temporal features of the neural data. Unless otherwise stated, we used the values of *T*_*w*_ ranging from 100 to 500 ms and those of *b* from 1 or 10 ms. Our task is to find all time windows that contain similar activity vectors in the same temporal order (Figures 2Ci–iv).

To make the N-W algorithm applicable to spike data, we make three extensions: scoring with the inner product, exponential gap penalty, and the local alignment of starting points. First, we define the degree of similarity between activity vectors observed in different time windows. In the comparison of two gene sequences, how to count the number of coincident letters (i.e., nucleotide bases) between the two sequences was naturally defined. However, the same scoring scheme is not applicable to neural activity data because neural population *in vivo* will hardly repeat exactly the same patterns due to various noise sources. In the extended N-W algorithm, we replaced delta function δ(*W*(1)[*j*], *W*(2)[*i*]) in the N-W algorithm with the inner product of activity vectors. Let matrices *W*(*t*_*k*_) and $W\left(t_{k^{\prime }}\right)$ be spiking activities of *N* neurons in the windows starting at time *t*_*k*_ and $t_{k^{\prime }}$, and **r**_*i*_(*t*_*k*_) and ${\text{r}}_{i}\left(t_{k^{\prime }}\right)$ be the column vectors in the *i-*th bin of *W*(*t*_*k*_) and $W\left(t_{k^{\prime }}\right)$, respectively (see Figure 2A):

(2) $$\begin{array}{lll} W\left(t_{k}\right) & = & \left({\text{r}}_{1}\left(t_{k}\right),{\text{r}}_{2}\left(t_{k}\right),\ldots ,{\text{r}}_{L}\left(t_{k}\right)\right), \end{array}$$

(3) $$\begin{array}{lll} W\left(t_{k^{\prime }}\right) & = & \left({\text{r}}_{1}\left(t_{k^{\prime }}\right),{\text{r}}_{2}\left(t_{k^{\prime }}\right),\ldots ,{\text{r}}_{L}\left(t_{k^{\prime }}\right)\right). \end{array}$$

Regarding *W*(*t*_*k*_) and **r**_*i*_(*t*_*k*_) (similarly for $W\left(t_{k^{\prime }}\right)$ and ${\text{r}}_{i}\left(t_{k^{\prime }}\right)$) as a string and a character in N-W algorithm, respectively, we measured the similarity between activity patterns at *t*_*k*_ and $t_{k^{\prime }}$ by the inner product ${\text{r}}_{i}\left(t_{k}\right)\cdot {\text{r}}_{j}\left(t_{k^{\prime }}\right)$.

Second, we developed a scoring scheme with exponential gap penalty, which penalizes edit similarity scores with an exponential discount factor when consecutive matches occur with different time lags in the two sequences compared, which is in spirit similar to the well-known linear gap penalty scheme (Gotoh, 1982). Below, two symbols $\upsilon_{j}^{i}$ and $\rho_{j}^{i}$ represent the optimal numbers of vertical gap insertion and horizontal gap insertion required for partial comparison up to the *i-*th element of *W*(*t*_*k*_) and the *j-*th element of *W*(*t*_*k*_), respectively. By inserting an additional gap, we may earn another coincidence at the cost of an additional discount factor in the similarity. Whether one should stop or continue the insertion of a gap is determined by the comparison of the cost and benefit of the two operations. To optimize the cost-benefit balance, we should insert a maximal number of gaps that do not cost more than the benefit. We set the initial conditions $\upsilon_{1}^{1:L+1}=\upsilon_{1:L+1}^{1}=\rho_{1}^{1:L+1}=\rho_{1:L+1}^{1}=0$ at the bottom row and the leftmost column of the table, where the notation $\upsilon_{1}^{1:L+1}=0$ means $\upsilon_{1}^{1},\;\upsilon_{1}^{2},\cdots \;,\;\upsilon_{1}^{L+1}=0$ and similarly $\upsilon_{1:L+1}^{1}=0$ means $\upsilon_{1}^{1},\;\upsilon_{2}^{1},\cdots \;,\;\upsilon_{L+1}^{1}=0$, and so on. Then, we calculate the values of $\upsilon_{j}^{i}$ and $\rho_{j}^{i}$ using the following recursive formula

(4) $$\begin{array}{l} \upsilon_{j}^{i}=\{\begin{array}{cc} 1 & \epsilon_{j}^{i-1}-\exp\left(\alpha \right)\geq \epsilon_{j}^{i-1-\upsilon_{j}^{i-1}}-\exp\left(\alpha \upsilon_{j}^{i-1}\right)\\ \upsilon_{j}^{i-1}+1 & \text{otherwise} \end{array}, \end{array}$$

(5) $$\begin{array}{l} \rho_{j}^{i}=\{\begin{array}{cc} 1 & \epsilon_{j-1}^{i}-\exp\left(\alpha \right)\geq \epsilon_{j-1-\rho_{j-1}^{i}}^{i}-\exp\left(\alpha \rho_{j-1}^{i}\right)\\ \rho_{j-1}^{i}+1 & \text{otherwise} \end{array}, \end{array}$$

where α is a free parameter to set the weight for the gap penalty, more specifically, the value of α determines the tolerance for the time lag between consecutive matches in the two-time windows. If α = 0.1, the penalty on the similarity score is –1 for a time lag of about 7.5. For the bin size of 1 ms, this value corresponds to a 7.5 ms-time difference between consecutive matches in the two windows. For instance, in the analysis of hippocampal data, which may contain relatively large jitters in spike times, the value of α was determined such that the time lag of 10 ms approximately yields the penalty of –1. The conditions to have $\upsilon_{j}^{i}=1$ and $\rho_{j}^{i}=1$ in the above equations are satisfied if the cost exceeds the benefit, and then we stop insertion of a gap. The values of $\upsilon_{j}^{i}$ and $\rho_{j}^{i}$ are calculated before $\epsilon_{j}^{i}$ in each cell.

Third, we solved the local alignment problem by applying the previously proposed algorithm (Smith and Waterman, 1981). In the case of strings (Figure 1), the heads of strings from which we start the comparison are obvious. However, the heads of cell assembly sequences are not given a priori in neural data. In our scheme, when no significant coincidences are found up to cell (*i, j*) and the score in that cell is below 0, we restart the recursive evaluation by setting $\epsilon_{j}^{i}$ to 0. In other words, we can jump from the bottom left cell to an arbitrary cell. This scheme results in the automatic search of the optimal starting points of the comparison.

In sum, recursive equation in N-W algorithm is changed into the following rule:

(6) $$\begin{array}{l} \epsilon_{j}^{i}=\max\{\begin{array}{c} 0\\ \epsilon_{j}^{i-\upsilon_{j}^{i-1}}-\exp\left(\alpha \upsilon_{j}^{i-1}\right)\\ \epsilon_{j-\rho_{j-1}^{i}}^{i}-\exp\left(\alpha \rho_{j-1}^{i}\right)\\ \epsilon_{j-1}^{i-1}+{\text{r}}_{i}\left(t_{k}\right)\cdot {\text{r}}_{j}\left(t_{k^{\prime }}\right) \end{array}, \end{array}$$

which is evaluated along with $\upsilon_{j}^{i}$ and $\rho_{j}^{i}$. Initial conditions are given as

(7) $$\begin{array}{l} \epsilon_{1}^{1},\;\epsilon_{2}^{1},\cdots \epsilon_{L}^{1}=0,\;\epsilon_{1}^{1},\;\epsilon_{1}^{2},\;\cdots \epsilon_{1}^{L}=0, \end{array}$$

and $\epsilon_{L+1}^{L+1}$ gives the maximum coincidence between the activity sequences, that is, the edit similarity score as in the standard N-W algorithm.

### 2.3. Metric Space and Two Algorithms for Clustering of Neural Data

Neural activity data segmented into different time windows form a high dimensional metric space in which edit similarity score defines a metric among data points. Namely, from edit similarity scores *E*(*k, k*′) between pairs of time windows *W*(*t*_*k*_) and $W\left(t_{k^{\prime }}\right)$, we can calculate a distance matrix as *D*(*k, k*′) = max(*E*(*k, k*′)) − *E*(*k, k*′), where the maximum is taken over all possible pairs of segments. In the high-dimensional feature space, time windows containing similar activity patterns are distributed at neighboring locations. Therefore, we can extract similar cell-assemblies through the clustering of data points. While similar activity patterns give a dense cluster, time windows containing no repeated patterns are scattered over the feature space as outliers. To remove these “noisy” components, we sequentially applied two qualitatively different types of clustering algorithms.

The first algorithm is called “OPTICS” and is a density-based clustering method. This algorithm aims to find dense clusters of data points (Ankerst et al., 1999). However, the algorithm cannot discriminate two clusters if they share a non-negligible number of data points. To overcome this weak point, we subsequently applied a second algorithm “COPRA,” which performs clustering based on a community detection scheme (Gregory, 2010). In short, the data points connected with relatively short distances (large edit similarities) are distinguished from other data points as a community in the feature space. In this study, we first applied OPTICS to remove noise from the data. The algorithm returns data indices for tentative clusters containing more than MinPts similar data points. Secondly, we applied COPRA to the extracted data points to fix their clustering labels.

### 2.4. Dimensionality Reduction by t-SNE

In **Figure 7A**, we visualized the results of clustering in a two-dimensional space using t-Distributed Stochastic Neighbor Embedding (t-SNE) (Maaten and Hinton, 2008). t-SNE is an algorithm that maps high-dimensional data into a low dimensional space, typically a two or three-dimensional space, while maintaining the original data structure in the high-dimensional manifold. In t-SNE, the similarity values are converted to the following conditional probability

(8) $$\begin{array}{l} p_{i|j}=\frac{\exp\left(-D\left(i,j\right)/\sigma_{i}\right)}{\sum_{k\neq i}\exp\left(-D\left(i,k\right)/\sigma_{i}\right)} \end{array}$$

and the joint probability is calculated by

(9) $$\begin{array}{l} p_{ij}=\frac{p_{i|j}+p_{j|i}}{2n} \end{array}$$

where *n* is the number of data points. In Equation (8), the parameter σ_*i*_ is modified to tune the visualization effect. We also modeled the joint probability of points *y*_*i*_ and *y*_*j*_ in a low-dimensional embedding space by using a Student t-distribution with one degree of freedom (also known as Cauchy distribution):

(10) $$\begin{array}{l} q_{ij}=\frac{{\left(1+\|y_{i}-y_{j}{\|}^{2}\right)}^{-1}}{\sum_{k\neq l}{\left(1+\|y_{k}-y_{l}{\|}^{2}\right)}^{-1}} \end{array}$$

Note that we set *p*_*i*|*i*_ and *q*_*i*|*i*_ to zero as we are only interested in pair wise similarity. The algorithm accomplishes a mapping by reducing the Kullback-Leibler divergence between *p*_*ij*_ and *q*_*ij*_

(11) $$\begin{array}{l} KL\left(P\|Q\right)=\underset{i\neq j}{\sum }p_{ij}\log\left(\frac{p_{ij}}{q_{ij}}\right), \end{array}$$

by using the gradient descent.

### 2.5. Tricks for Reducing the Computational Cost

The proposed method, in its original form, requires extensive computational resources when used on neural data. The major difficulty comes from the calculation of a similarity matrix that has a computational complexity of *O*(*M*^2^), where *M* is the number of time windows and grows with the data length *T*. The number of window pairs easily becomes astronomically large even for relatively short data. For instance, if *T* = 20 min and the size of non-overlapping time windows is 100 ms, the number of window pairs is 1.44 × 10^8^. Because we cannot practically calculate similarity scores for all possible window pairs, we accelerated the calculation of edit similarity drastically by employing an approximation algorithm based on the Jaccard similarity (Cohen et al., 2001). For instance, with this algorithm we needed to calculate the similarity scores for 3 percent of window pairs in the hippocampal data analyzed later, meaning that the method reduced the computation time by approximately 97%. Note that an exact computation without using the Jaccard similarity was not attempted due to unrealistically long computation time.

In this procedure, we reduce the calculation of edit similarity for pairs of time windows that do not share active neurons. Let (w_*i*_(*t*_*k*_)) be a Boolean matrix in which the element (*i, k*) is 1 if neuron *i* fires at least once in the time window at *t*_*k*_ or otherwise 0:

(12) $$\begin{array}{l} w_{i}\left(t_{k}\right)=\{\begin{array}{cc} 1 & \sum_{b=0}^{L}\left(W_{b}^{i}\left(t_{k}\right)\right)\geq 1\\ 0 & \text{otherwise} \end{array}\;, \end{array}$$

where $W_{b}^{i}\left(t_{k}\right)$ is the (*i, b*) element of *W*(*t*_*k*_). We measure the similarity between **w**(*t*_*k*_) and $\text{w}\left(t_{k^{\prime }}\right)$ by Jaccard similarity defined as

(13) $$\begin{array}{l} \text{Jaccard}\left(\text{w}\left(t_{k}\right),\text{w}\left(t_{k^{\prime }}\right)\right)=\frac{\left|\text{w}\left(t_{k}\right)\cap \text{w}\left(t_{k^{\prime }}\right)\right|}{\left|\text{w}\left(t_{k}\right)\cup \text{w}\left(t_{k^{\prime }}\right)\right|},\;\left(k,\;k^{\prime }=1,\cdots \;,N_{w}\right) \end{array}$$

where |**x** ∩ **y**| denotes the inner product of given two vectors, |**x** ∪ **y**| counts number of non-zero elements of the sum of them. The value of Jaccard similarity is between 0 and 1, and is close to unity if the column vectors at time *t*_*k*_ and $t_{k^{\prime }}$ are similar.

Because the calculation of Jaccard similarity for every possible pair of vectors is also *O*(*M*^2^), we wish to find out pairs that are likely to give highly similar **w**(*t*_*k*_) without direct calculation. For this purpose, we can make use of the statistical properties of Jaccard similarity. Now a trick is to use hash function $\tilde{h}\left(\text{x}\right)$ which assigns a different integer to a given integer *x* without collision: the hash function should not return the same integer to different values of *x*. Throughout this study, we used a built-in hash function of programming language Julia (https://julialang.org/). For a vector of integers, we defined the function $h\left(\text{x}\right)=\min\tilde{h}\left({x^{\prime }}_{i}\right),\;x_{i}^{\prime }\in \text{x},\;{x^{\prime }}_{i}\neq 0$, which returns the minimum of the hashed numbers assigned to non-zero elements of **x**. The value is called the minimum hash (min-hash) value. Importantly, the following relationship holds (Cohen et al., 2001):

(14) $$\begin{array}{l} \text{Prob}\left[h\left(\text{w}\left(t_{k}\right)\right)=h\left(\text{w}\left(t_{k^{\prime }}\right)\right)\right]=\frac{\left|\text{w}\left(t_{k}\right)\cap \text{w}\left(t_{k^{\prime }}\right)\right|}{\left|\text{w}\left(t_{k}\right)\cup \text{w}\left(t_{k^{\prime }}\right)\right|}\\ \;=\text{Jaccard}\left(\text{w}\left(t_{k}\right),\text{w}\left(t_{k^{\prime }}\right)\right) \end{array}$$

With this relationship, we can obtain Jaccard similarity without pair-wise comparisons of column vectors:

(15) $$\begin{array}{l} \text{Jaccard}\left(\tilde{\text{w}}\left(t_{k}\right),\tilde{\text{w}}\left(t_{k^{\prime }}\right)\right)\approx \frac{\left|\left\{q|1\leq q\leq n\;\text{and }{\tilde{S}}_{k}^{q}={\tilde{S}}_{k^{\prime }}^{q}\right\}\right|}{n} \end{array}$$

where $\tilde{S}$ is called a signature matrix that contains the min-hash values over different random permutations of $\tilde{\text{w}}\left(t_{k}\right)$, i.e., ${\tilde{S}}_{k}^{q}=h_{q}\left(\tilde{\text{w}}\left(t_{k}\right)\right)$ with *h*_*q*_ being the *q*-th min-hash function. The total number *n* of random permutations is dynamically adjusted as explained in the next section. In this matrix, elements in a column are min-hash values of a time window generated with different hash functions, and elements in a row are min-hash values of all time windows generated with a hash function.

**Figure 3:**
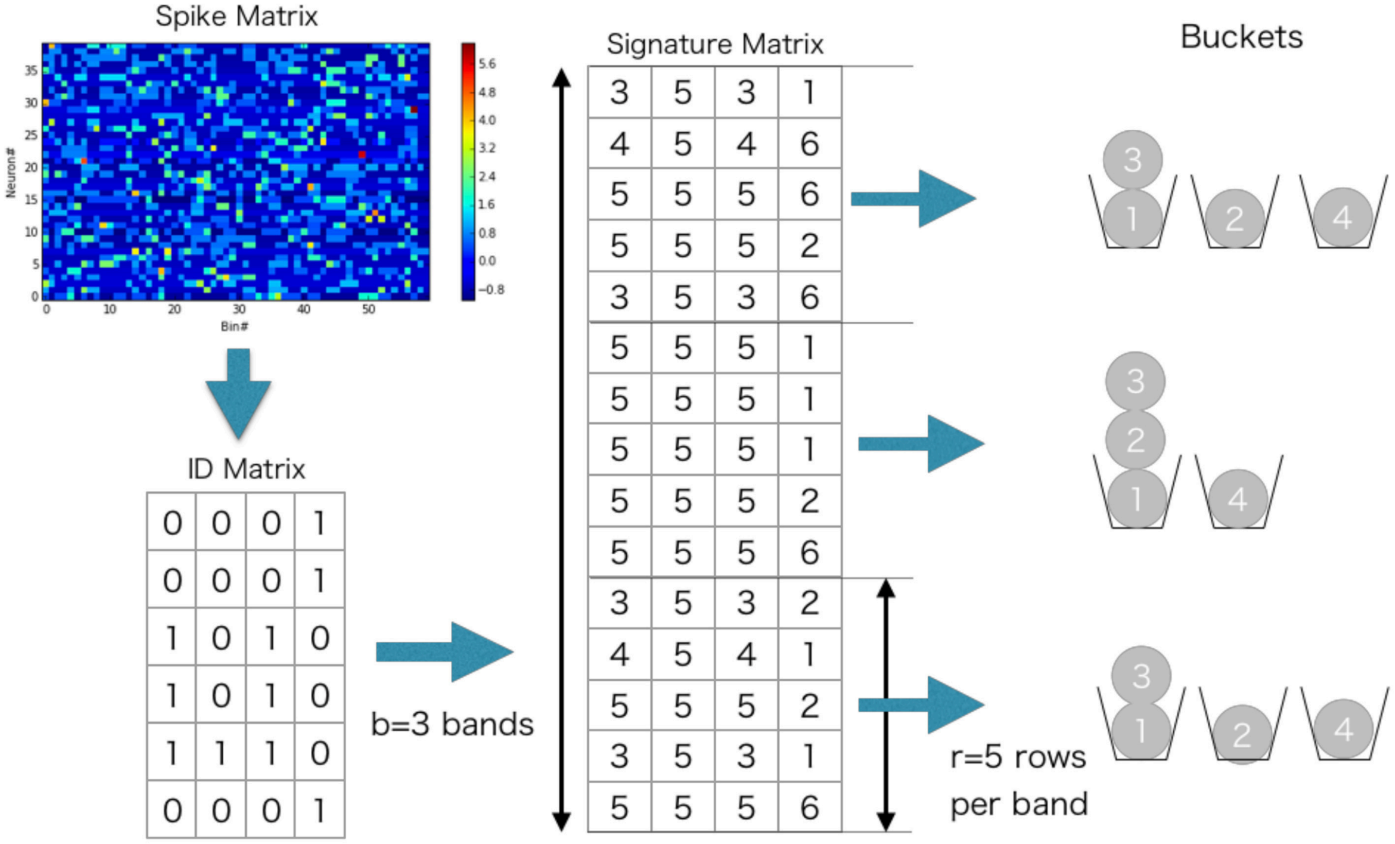
Minhashing procedure illustrated.

To further reduce computation, we used the following banding technique in the evaluation of Jaccard similarity (Cohen et al., 2001) (Example can be found in Figure 3). We divided $\tilde{S}$ into *b* bands of *l* rows each, thus *n* = *bl*. Suppose that two vectors $\tilde{\text{w}}\left(t_{k}\right)$ and $\tilde{\text{w}}\left(t_{k^{\prime }}\right)$ have Jaccard similarity *s*, then the probability that the min-hash signatures of two columns coincide at least in one row of the matrix is *s*. Then, the probability that the signatures of two columns are identical in all rows of at least one band is *p*(*s*) = 1 − (1 − *s*^*l*^)^*b*^, which is an S-shaped function of *s* and can hence be used for determining a threshold value of the similarity. We hash all the bands, and search bands in which two columns have the same hash value. The only pairs of time windows that have the same hash value in more than one band are used for similarity matrix calculation. For instance, *p*(0) = 0.000, *p*(0.3) = 0.007, *p*(0.5) = 0.091, *p*(0.7) = 0.424, *p*(0.8) = 0.696, *p*(0.9) = 0.931, and *p*(1.0) = 1.000 when *b* = 5 and *l* = 2. In the present analysis, the values of *b* and *l* were dynamically adjusted by data itself. We explain the method for the adjustment in the next section.

### 2.6. A Policy for Division of Signature Matrix

To apply the above algorithm to neural data, we employed a heuristic method to determine the two parameters *b* and *l* for Jaccard similarity from neural data. The aim of this method is to reduce the load of heavy computation for large neural data without losing candidate sequences. We calculated the average firing rates of individual neurons over the entire length of data, and we determined *b* and *l* assuming independent Poisson spiking neurons having the same firing rates. The parameters (*b* and *l*) for a smaller threshold (Jaccard_1_) gives the similarity expected under the assumption of independent Poisson spiking, whereas a parameter for a larger threshold (Jaccard_2_) represents the similarity expected when the two time windows contain sequences with a certain length. Let #_*i*_ be the total number of spikes of neurons *i* during the interval [0, *T*]. From #_*i*_, we can calculate the probability that a neuron *i* has at least one spike in the segment *W*(*t*_*k*_) as $p_{i}=1-{\left(1-\left({\#}_{i}/T\right)\Delta \right)}^{\left(L/\Delta \right)}$, where Δ is the size of a bin. Then, the index $N_{1}=\overset{N}{\underset{i=1}{\sum }}p_{i}$ is the expected number of active neurons within the time window. Then, the expected number of coincidently active neurons in an arbitrary pair of time windows is $N_{2}=\overset{N}{\underset{i=1}{\sum }}{\left(p_{i}\right)}^{2}$, and Jaccard_1_ is calculated as *N*_2_/(2*N*_1_ − *N*_2_).

Now, suppose that two time windows contain additional *N*_3_ coincidently active neurons. In this case, the expected Jaccard similarity, or Jaccard_2_, is given as (*N*_2_ + *N*_3_)/(2*N*_1_ − *N*_2_ − *N*_3_). In this study, we searched values such as *b* and *l* that keep the probability 1 − (1 − *s*^*l*^)^*b*^ sufficiently high (e.g., 0.8) for Jaccard_1_ and sufficiently low (e.g., 0.1) for Jaccard_2_. The parameters *b* and *l* were searched in a brute-force manner. In this paper, *b* and *l* were searched in [1, 50].

### 2.7. Validity of Our Method for Similarity Matrix Calculation

We examined whether the method Cohen et al. (2001) facilitates faster computation by selecting high-similarity window pairs.

**Figure 4:**
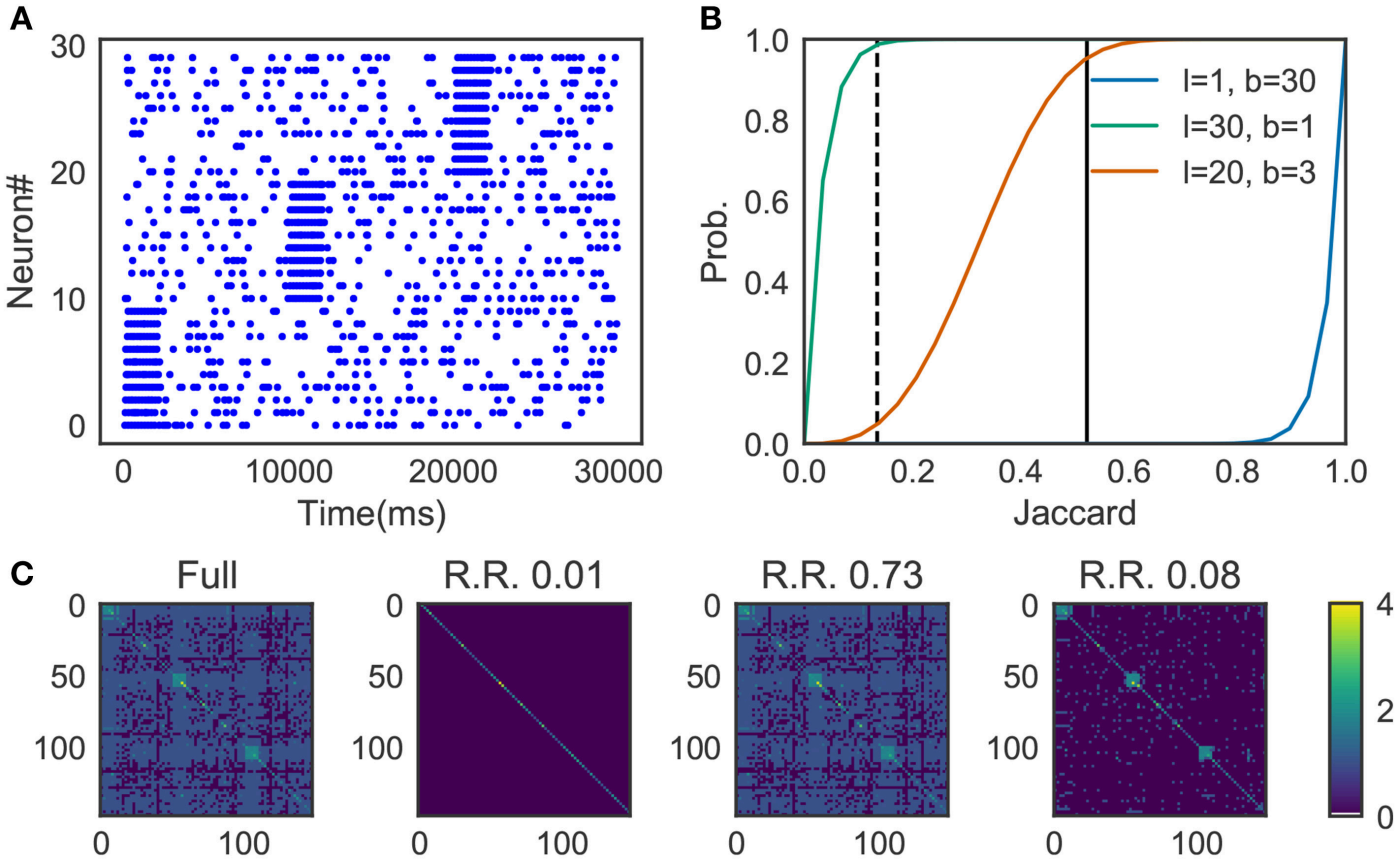
Parameter-dependence of sequence detection. **(A)** Synthetic data used for the evaluation which contain three different sequence patterns ten times each. **(B)** The similarity matrices were compared between three different parameter sets for Cohen et al. (2001). The dotted line and thick black line show Jaccard_1_ and Jaccard_2_, respectively. When the number of bands is small and the size of the band is small (*l* = 30, *b* = 1, shown in green), similarity of all candidates was calculated. In contrast, with the large number of bands and the small band size (*l* = 1, *b* = 30) the algorithm discarded all the pairs. A parameter set should be chosen such that the algorithm can discard pairs corresponds to Jaccard_1_ and holds pairs of Jaccard_2_. The parameter was searched in a brute-force manner. **(C)** Similarity matrix was calculated without using the method in Cohen et al. (2001). With (*l* = 20, *b* = 3), only 8% of the (row, col)-pairs calculated (shown as R.R., reduced rate) yet three clusters embedded in the data successfully detected.

We prepared synthetic data (Figure 4A). The data consists of three sequences of different cell assemblies, each of which appeared ten times in the data (each type of sequences appear consecutively for clear visualization). Random noise was generated by a 1 Hz Poisson process and was superimposed on the sequences. The parameter values used are described in 2.13.

Figure 4B shows the probability *p*(*s*) = 1 − (1 − *s*^*l*^)^*b*^, where the dashed line refers to the Jaccard_1_ and the thick black line to the Jaccard_2_. As we can see, only the parameter set *l* = 20, *b* = 3 provides the appropriate condition, that is, high probability in Jaccard_1_ and low probability in Jaccard_2_.

Figure 4C shows four similarity matrices with and without the method. If you prepare too many hash functions and put all of them into a single band (the second from the left. *l* = 30, *b* = 1 which corresponds to the green line in panel B) the algorithm ignores almost all pairs. Also, too much banding causes another problem; the algorithm cannot ignore less similar pairs. That is the case described in the third from the left (*l* = 1, *b* = 30 which corresponds to the blue line in panel B). With the appropriate parameter set (*l* = 20, *b* = 3, the orange line in panel B), our method successfully detected three embedded clusters although only about 8% of elements were calculated (the rightmost case).

### 2.8. Construction of Profiles for Clustered Sequences

Because activity patterns belonging to a cluster in general exhibit a large variation in the temporal structure, a method was necessary to identify the core temporal structure of the cell assembly sequences belonging to each cluster. Here, we explain our iterative multiple alignment algorithm for constructing profiles of clusters. It is based on the algorithm by Barton and Sternberg (1987). In the original algorithm, we initialize the algorithm with a tentative profile, which is obtained by taking the longest common subsequence between the two time windows in a cluster that show the highest match in edit similarity. After the initialization, we search the next time window that gives the most similar profile to the tentative one, and update the tentative profile using edit similarity. We repeat this procedure until the tentative profile converges.

In our method, we made two major modifications to the original algorithm. First, we chose two arbitrary time windows in the initiation step to reduce the computational cost. The final results did not significantly differ between our approach and the original one. As shown in (**Figure 11A**), both original and simplified methods generated similar profiles for the data used in section 3.3. Second, in generating a profile, we used the instantaneous value of z-score of spike count in each time window. Namely, for each neuron, we calculated the average and variance of spike count per bin over the entire data, and then subtracted the average from spike count in each bin and normalized the difference by the variance. The use of z-score suppresses the influences of highly active neurons on the detection of ensemble firing sequences. Finally, in each step, a Gaussian filter with mean 0 and variance σ was applied to the tentative profile. Variance σ was initially as large as the window size and gradually reduced to the bin size as iterations proceeded. This filtering prevented a profile from containing more than one similar sequence, thus enabled a robust detection of minimal sequences.

### 2.9. *F*_S_-Score for Supervised Clustering

Performance of supervised cell-assembly detection from artificial data was scored in terms of *F*_S_-score, which is given as the harmonic mean of Precision and Recall:

(16) $$\begin{array}{l} F_{\text{S}}=2{\left(\frac{1}{\text{Precision}}+\frac{1}{\text{Recall}}\right)}^{-1}. \end{array}$$

where Precision and Recall are defined as

(17) $$\begin{array}{l} \text{Precision}=\frac{\text{TP}}{\text{TP}+\text{FP}}, \end{array}$$

(18) $$\begin{array}{l} \text{Recall}=\frac{\text{TP}}{\text{TP}+\text{FN}}, \end{array}$$

in terms of true positives (TP), false positives (FP) and false negatives (FN). We also used Specificity, which is defined as

(19) $$\begin{array}{l} \text{Specificity}=\frac{\text{TN}}{\text{TN}+\text{FP}}, \end{array}$$

to evaluate the portion of negatives that are correctly classified as such.

### 2.10. *F*_US_-Score for Unsupervised Clustering

Performance of unsupervised cell-assembly detection of artificial data was scored in terms of *F*_US_-score, which is widely used for unsupervised clustering in the field of machine learning (c.f. Artiles et al., 2007). The score is given as the harmonic mean of Purity and Inverse Purity as

(20) $$\begin{array}{l} F_{\text{US}}=2{\left(\frac{1}{\text{Purity}}+\frac{1}{\text{Inverse Purity}}\right)}^{-1}. \end{array}$$

We note that this score is different from *F*_S_-score for supervised clustering. Purity is a weighted average of the fractions of true members in detected clusters,

(21) $$\begin{array}{l} \text{Purity}=\overset{m}{\underset{i=1}{\sum }}\frac{\left|C_{i}\right|}{T}\frac{C_{i}\cap L_{j}}{C_{i}}, \end{array}$$

and Inverse Purity is a weighted average of correctly classified portions of true clusters,

(22) $$\begin{array}{l} \text{Inverse Purity}=\overset{n}{\underset{i=1}{\sum }}\frac{\left|L_{i}\right|}{T}\frac{C_{i}\cap L_{j}}{L_{j}}, \end{array}$$

where *m* is the number of detected clusters *C* = {*C*_1_, *C*_2_, …, *C*_*m*_}, *n* is the number of true clusters plus a noise cluster in the artificial data *L* = {*L*_1_, *L*_2_, …, *L*_*n*_}, and *T* is the total number of data points (i.e., time windows). The noise cluster consists of spurious cell assemblies. (*C*_*i*_ ∩ *L*_*j*_/*C*_*i*_) represents the fraction of members of the *j*-th true cluster in the *i*-th detected cluster. *j*-th true cluster is selected by argmax_*k*_*C*_*i*_ ∩ *L*_*k*_. In the above expressions, weights are determined such that a larger cluster contributes more strongly to the weighted sums. We note that Purity and Inverse Purity take their values within the interval [0, 1]. If a classification is perfect, both Purity and Inverse Purity take the maximal value of unity. The harmonic mean of Purity and Inverse Purity penalizes two trivial solutions. In one such solution each data point constitutes an independent cluster (i.e., *m* = *T*), and in the other solution all data points are classified into a large cluster. In these trivial cases, Purity, but not Inverse Purity, takes the maximum value of unity.

### 2.11. Cluster Labels for PCA/ICA-Based Analyses

To compare our method with the PCA/ICA-based methods for detecting synchronously firing ensembles (Lopes-dos Santos et al., 2013), we have to assign a cluster label to each component detected by these methods. To this end, we calculated overlaps between the population activity vector and the principal (or independent) components for all time windows. Then, in each time window, the component having the highest overlap with the instantaneous population activity was assigned to the time window as the corresponding cluster label. If the highest overlap in a time window did not reach a certain threshold, no PCA/ICA components were assigned to the window and this time window was treated as noise in the calculation of *F*_US_-score. The above procedure was repeated until all time windows were labeled with some PCA/ICA components or classified as noise. For a fair comparison with our method, an optimal threshold value that maximizes the *F*_US_-score was searched in brute-force manner.

### 2.12. Behavioral Labels for Clusters

We introduced three behavioral labels (Go, Back, Stop) in the analysis of clusters of hippocampal neurons. First, individual members (time windows) of each cluster were labeled with “Go,” “Back,” or “Stop” according to the directions and average speed of animal's movement in the corresponding time window. A member was labeled as “Stop” if the average speed was less than 3 cm/s, and Go refers to the movement direction from start to goal and Back refers to the opposite direction. Then, in each cluster we constructed gaussian kernel density functions (KDF) to fit the spatial distributions of cluster members labeled with the different behavioral labels and measured the peak heights of these KDFs. Now, for each behavioral label we selected the top 20 clusters yielding the highest peaks of the corresponding KDF. We called thus-obtained three sets of 20 clusters as “Go cluster,” “Back cluster,” and “Stop cluster” in **Figure 9A**. We note that this categorization scheme allows a cluster of time windows to obtain multiple behavioral labels.

### 2.13. Parameter Choices

Here we list the values of parameters in our algorithm.

In our approximate calculations of similarity matrix (Cohen et al., 2001) in section 2.7, we used the following parameters: the length of sliding time window *T*_*w*_ = 200, the penalty parameter α = 0.1.

We searched the set of parameter values that maximizes the detection performance in **Figure 6A**. Each parameter was tested within the following range: the number of points for a minimal cluster in OPTICS MinPts from 2 to 20 (Ankerst et al., 1999); parameter for COPRA *v* from 2 to 20 (Gregory, 2010); the parameter α = 1.0; MinPts = 5 and v = 5 in **Figures 6F,G**.

For the hippocampal data, the following parameter values were used: the parameter α = 0.1; the length of sliding time window *T*_*w*_ = 100ms, which is close to the period of one cycle of theta oscillation; parameter for Jaccard_2_ *N*_3_ = 10; the number of points for a minimal cluster in OPTICS MinPts = 20; parameter for COPRA *v* = 4.

The prefrontal data were analyzed using sliding time windows with a wider variety of lengths ranging from 250 ms to 2.5 s because the characteristic time scale of sequences was not known. However, all the results shown in this study were obtained for the length of 250 ms. The temporal discount factor was set as α = 0.03. Other parameters were as follows: parameter for Jaccard_2_ *N*_3_ = 10; the number of points for a minimal cluster in OPTICS MinPts = 400; parameter for COPRA *v* = 30.

### 2.14. Bayesian Modeling for Edit Similarity Difference

In the analysis of hippocampal activity, we searched locations in a linear maze at which the recorded neural activity coincides with a detected cell assembly in a statistically meaningful manner. We divided the linear maze (its total length ranged from 60 to 300 cm) into 30 different locations which we may term position bins. In each position bin (denoted as *x*), we computed an edit similarity score ${\text{ED}}_{\text{x}}^{\text{raw}}$ between the profile of the given cell assembly and neural activity. Similarly, we calculated an edit similarity score ${\text{ED}}_{\text{x}}^{\text{sge}}$ for surrogate data in which spikes of each neuron were randomly shuffled across time bins (this corresponds to the null model of homogeneous Poisson processes). Then, we used Bayesian modeling for estimating the significance of similarity score at each position bin compared with the null model. Let μ_*x*_ be the baseline score at the position bin *x*. We assumed that the value of μ_*x*_ smoothly changes across positions until the score exhibits a sudden jump at some position bins. To be specific, we assumed that ${\text{ED}}_{\text{x}}^{\text{raw}}$ and ${\text{ED}}_{\text{x}}^{\text{sge}}$ obey the following Gaussian distributions:

$$\begin{array}{l} {\text{ED}}_{\text{x}}^{\text{raw}}\sim \text{Normal}\left(\mu_{\text{x}},\sigma^{\text{raw}}\right),\;{\text{ED}}_{\text{x}}^{\text{sge}}\sim \text{Normal}\left(\mu_{\text{x}}+\delta_{\text{x}},\sigma^{\text{sge}}\right). \end{array}$$

Then, we assumed that the change μ_*x*_ − μ_*x*−1_ obeys the Gaussian distribution with the mean μ_*x*−1_ − μ_*x*−2_ and the variance σ_μ_ and that a jump δ_*x*_ obeys the Cauchy distribution with the mean δ_*x*−1_ and the variance σ_δ_:

$$\begin{array}{l} \mu_{x}\sim \text{Normal}\left(2\mu_{\text{x}-1}-\mu_{\text{x}-2},\sigma_{\mu }\right),\;\delta_{\text{x}}\sim \text{Cauchy}\left(\delta_{\text{x}-1},\sigma_{\delta }\right). \end{array}$$

For the statistical modeling, we used STAN library (http://mc-stan.org/) with default uniform prior distributions for the variances σ_μ_, σ_δ_, σ^raw^ and σ^sge^.

### 2.15. Code Accessibility

Data analysis was done by Python 3.6, Julia 0.6, and Bash. The implementation of the algorithm in Python 3.6 is available at (https://github.com/KeitaW/spikesim, RRID: SCR_016351). Only part of the algorithms related to calculating the spike similarity is provided in the source code on the repository. We used the original implementation of COPRA (Gregory, 2010) and OPTICS (Zhang et al., 2013) We also used GNU parallel (Tange, 2011) for data processing.

### 2.16. Experimental Data

The data of hippocampal neurons is available at the data sharing website of Collaborative Research in Computational Neuroscience (CRCNS.org., http://dx.doi.org/10.6080/K09G5JRZ)(Mizuseki et al., 2009). The data used in this study contains the activity of 108 neurons recorded from the hippocampal CA1 region of a male Long-Evans rat during voluntary exploration of a linear maze. The total duration of recordings is 1928s. The multi-neuron spike trains of prefronal neurons used in this study were recorded previously from the medial prefrontal cortex of a male Brown Norway/Fisher hybrid rat with a chronically implanted hyper drive consisting of 12 tetrodes (Euston et al., 2007). The data contains the activity of 76 neurons and the total duration of recordings is 11, 010s.

## 3. Results

We developed a method for robust sequence detection based on the edit similarity score known in computer science (Levenshtein, 1966). The basic concept of edit similarity is simple. Suppose that we evaluate similarity between two strings of genes, “ATCGTAC” and “ATGTTAT.” We may naively count the number of coincident bases at the corresponding positions in the two strings. In the above example, the first two bases “AT” coincide, so the similarity is two. However, if we count the maximal number of coincidences preserving the serial orders of bases but allowing the insertion of blanks “-,” we may compare “ATCGT-A-C” and “AT-GTTAT-” to obtain the maximal number of five (i.e., A, T, G, T and A coincide in this order). The N-W algorithm (Needleman and Wunsch, 1970) provides a rigorous method for scoring edit similarity between arbitrary strings. In this study, we extended this algorithm such that it is applicable to neural data (section 2).

### 3.1. Density- and Community-Based Algorithms for Sequence Clustering

In our method, neural data are segmented into time windows and those containing similar sequential activity patterns are searched (see Figure 2A). These time windows form a high dimensional space with a metric defined by edit similarity score, and similar activity patterns form a cluster of neighboring data points (i.e., a candidate of cell assembly) in this feature space. To identify these clusters, we used a density-based clustering algorithm “OPTICS” (Ankerst et al., 1999) and a community-based clustering algorithm “COPRA” (Gregory, 2010) (see section 2).

**Figure 5:**
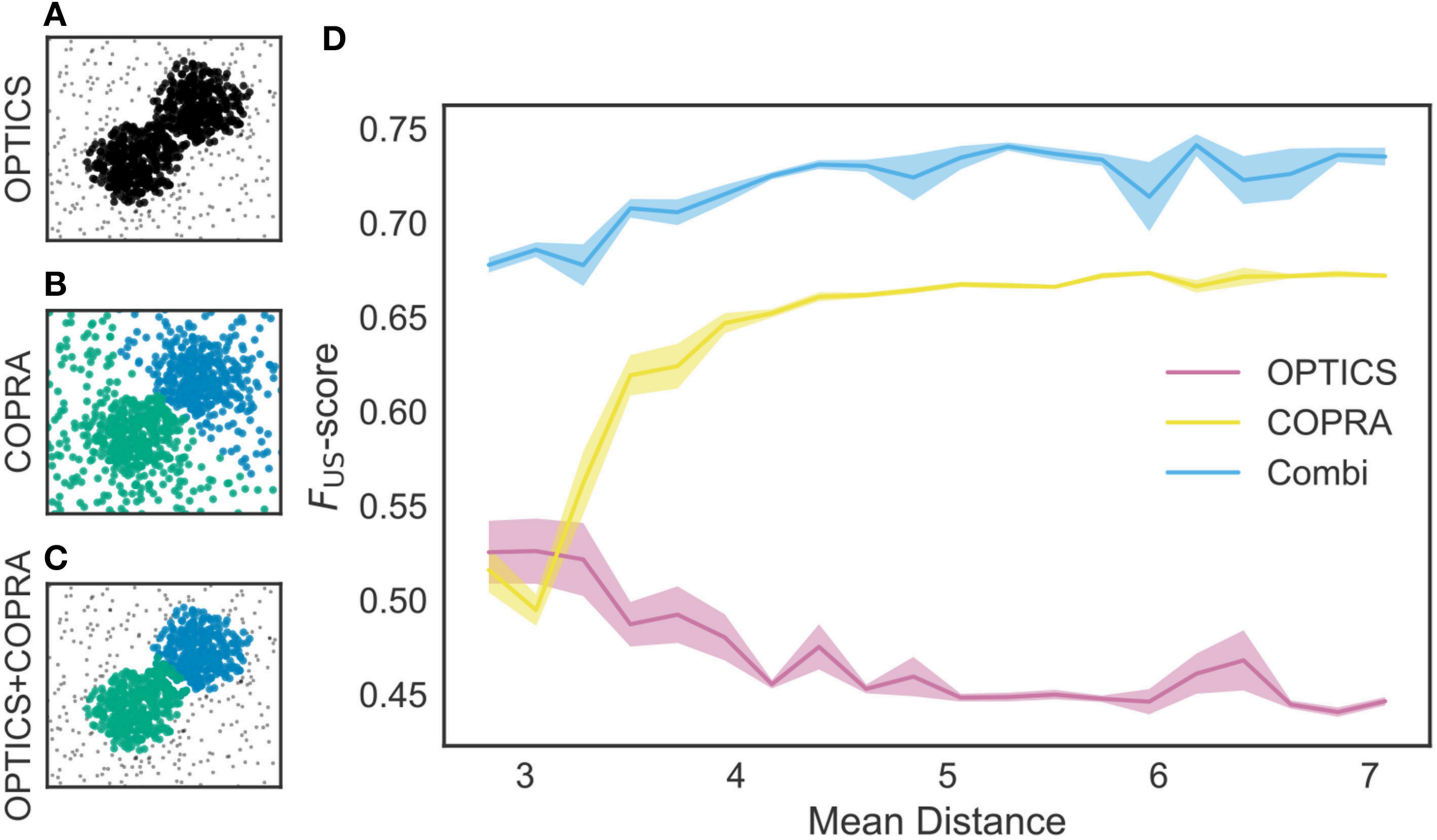
Comparison between different clustering algorithms. A density-based clustering algorithm (OPTICS) and a community detection algorithm (COPRA) were separately or sequentially applied to a dataset. Data points were generated by a mixture of two Gaussian distributions with different centers and the same variance of 1.3. These points were further mixed with uniformly distributed background data points. **(A)** OPTICS could remove background noise but failed to discriminate the two clusters. **(B)** COPRA could separate these clusters but failed to remove background noise. **(C)** The combined application of OPTICS and COPRA successfully separated the two clusters and removed background noise. **(D)** Performance of identifying two clusters was compared between the different algorithms, that is, OPTICS only (magenta), COPRA only (yellow) and their combination (cyan). The abscissa represents the distance between the centers of the two clusters. Shaded areas show standard errors.

We tested these methods using an artificial dataset. As shown in Figure 5A, OPTICS identified a single dense cluster from noisy data points, but it did not separate the cluster into two parts. On the other hand, “COPRA” identified two separate clusters but each cluster contained a considerable number of noisy data points: an outlier may be invited to a community if its distance from any member of the community is short enough (Figure 5B). In this study, we sequentially applied OPTICS and COPRA to take advantage of each method (Figure 5C). The combined use of the two algorithms compensated for the weakness of others to improve clustering performance. As studied in Figure 5D, the combined use was especially advantageous when the mean distance between data points was small. To our knowledge, such advantage has not previously been pointed out.

### 3.2. Performance Evaluation With Artificial Data

**Figure 6:**
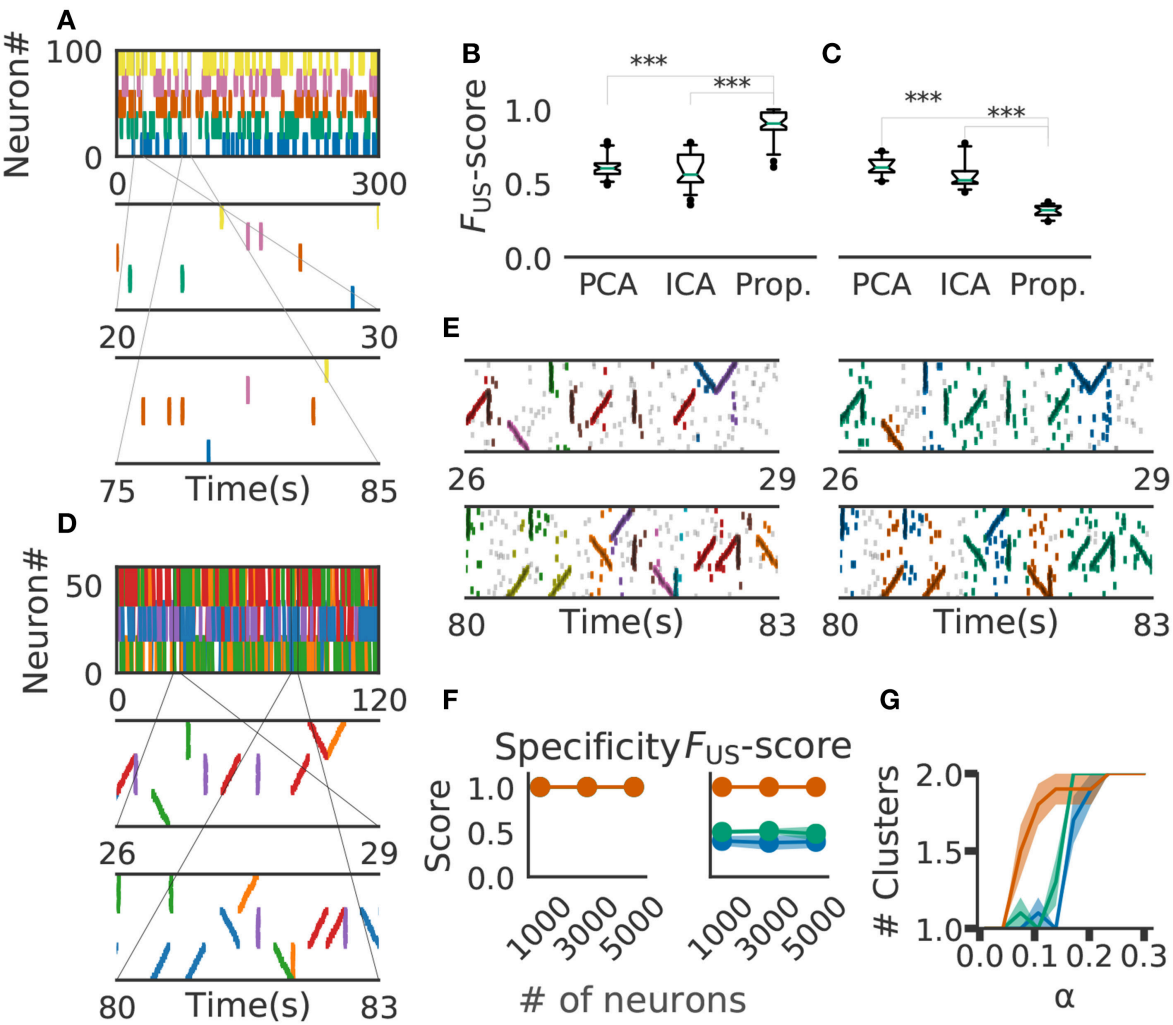
Comparison with PCA-/ICA-based methods. **(A)** An example of the embedded artificial cell assemblies used for the comparison. In the raster plot, each dot is a spike. Each color indicates a cell assembly. For clarity, noisy spikes are not shown. **(B)** Timing jitters were within ±10 ms and cell assemblies represented synchronously firing neuron ensembles. F_US_ -score was significantly higher for the proposed method than for PCA- and ICA-based algorithms (*p*-values were less than 2.2e-16 for both cases: Wilcoxon rank sum test). The time window used was 200 ms. **(C)** Timing jitters were within ±50 ms and cell assemblies may not be regarded as synchronously firing neuron ensembles. Our method is sensitive to the serial order of firing and hence the score is lowered (*p*-values were less than 2.9e-11 for both cases: Wilcoxon rank sum test). **(D)** Nine artificial spike sequences (middle and bottom) were embedded into noisy spike trains. Noise spikes are not shown here. **(E)** Sequences detected by our method from the artificial data shown in D are presented in two intervals together with noisy spikes (gray). The nine embedded sequences were successfully detected. The results are shown for different values of parameters: α = 2.0, MinPts = 20, v = 2 in left panels; α = 0.5, MinPts = 10, v = 12 in right panels. **(F)** Robustness of cell-assembly detection against background noise. Two scores, Specificity (left) and *F*_S_ (right), in the detection of a single cell-assembly are shown against the number of background Poisson spike trains: our method (orange), PCA (green), and ICA (blue). Shaded areas indicate standard errors. **(G)** Effect on different α for cluster formation. Different shrinkage rates tested: 10 (orange), 5 (green), and 3 (blue). Shaded areas indicate standard errors.

We compared the performance of our method with that of PCA- (Peyrache et al., 2009; Lopes-dos Santos et al., 2011) and ICA-based method (Lopes-dos Santos et al., 2013) by using synthetic population activity data. We embedded 5 non-overlapping cell assemblies into background activity of 100 simulated neurons firing independently at a rate of 2[Hz] (Figure 6A, top panel). PCA and ICA do not take the time structure of cell assemblies into account, but these methods should be good at detecting the assemblies of synchronously firing neurons. On the other hand, our method treats synchronously firing as a special case of sequential firing with zero time lags between spikes. In fact, we generally expect slightly better performance for synchronous firing than for sequential firing because the probability that the complete cell-assembly pattern falls within a time window will be higher for the former than for the latter. Therefore, we constructed synthetic data of length 300s in which each cell assembly consisted of 20 synchronously firing neurons with certain timing jitters and appeared 50 times at randomly determined positions. The same size of time window (200 ms) was used in all the methods and no spikes of each cell assembly occurred across different time windows.

For performance evaluation, we searched the set of parameter values that maximizes the detection performance. Each parameter was tested within the following range the number of points for a minimal cluster in OPTICS MinPts 2 to 20 (Ankerst et al., 1999); parameter for COPRA *v* 2 to 20 (Gregory, 2010). We evaluated the performance of each method in terms of *F*_US_-score (section 2). We generated 40 artificial data with different background activity. We then analyzed each data by the three methods (i.e., PCA-based, ICA-based and the proposed methods) and calculated *F*_US_-score for each trial. The resultant score was significantly larger in the proposal method (mean ± s.d., 0.89 ± 0.09) than in the PCA-based (0.61 ± 0.07) and ICA-based (0.59 ± 0.11) methods (Figure 6B). In fact, our method correctly detected all target cell assemblies.

In Figure 6B, the value of timing jitters is relatively small (±10 ms) and cell assemblies may be regarded as groups of synchronously firing neurons. For shorter timing jitters, the results would not change significantly. However, for longer jitters our method will exhibit degraded *F*_US_-score because the method is sensitive to the serial order of firing. In contrast, PCA/ICA-based methods do not take the order of firing into account and therefore will exhibit no significant differences, as far as neurons belonging to each cell assembly fire within the same time windows. We studied this large-jitter case by using timing jitters of ±50ms without changing the discount factor (i.e., α = 0.1) in Figure 6C. As expected, PCA/ICA-based methods show seemingly better performance compared to our method. These results indicate that our method is more sensitive to timing jitters than PCA-/ICA-based method. However, as we discuss in the next paragraph, sensitivity of our model can be controlled by changing α.

We then investigated if our method is able to extract spike sequences in noisy artificial data. Sequential firing of three non-overlapping cell assemblies each consisting of 20 neurons was embedded into background activity of 60 neurons (including the twenty) at a rate of 1Hz in both forward, synchronous and reverse orders with ±10 ms jitter (Figure 6D). Each sequence appeared 20 times. The time window was 200 ms and bin size was 10 ms. Our method detected the groups of cells firing sequentially as well as synchronously, but the way our method categorized these cells depended on the values of parameters used and noise level in input data. In the left panels of Figure 6E, the same group of neurons firing in different temporal orders were categorized as separate clusters (e.g., green and purple clusters). Namely, we can see three separate groups of cells that fire with an upward ramp, a downward ramp, or all simultaneously. In contrast, such a group of cells was classified into a single cluster in the right panels of Figure 6E. Although some spikes were misidentified, the following scores quantify the performance of our algorithm in detecting the three clusters: Precision = 0.75, Recall = 0.85, and the *F*_S_-score = 0.8 in Figure 6, left; Precision = 0.63, Recall = 0.89 and the *F*_S_-score = 0.73 in Figure 6E, right. The scores used are explained in section 2.

We further investigated the robustness of performance of our method at different signal-to-noise ratios. Here, we varied the ratio by modifying the number of background firing neurons. We embedded a single cell assembly consisting of 100 neurons firing synchronously without jitters into a sizable neural population while maintaining each neuron firing rate of 5 Hz. The cell-assembly pattern appeared 20 times at random temporal positions in neural activity data of the total length of 60 s. We generated ten instantiations for each total number of neurons ([1,000, 3,000, 5,000]). We then analyzed these data sets by our method with the time windows of 200 ms and calculated *F*_S_-score for supervised clustering for each set (section 2). The results are shown in Figure 6F, which proves the robustness of performance against changes in the magnitude of background noise. We used the parameter α = 1.0, MinPts 5 and v 5 in this analysis.

Finally, we tested that the method's ability to detect a cluster of assembly sequences that were activated on two different time scales. Each assembly activation consists of 30 different neurons which were sequentially activated in 200 ms and embedded 20 times. In addition, we added the same numbers of compressed patterns. We used three different shrinkage factors, three, five, and ten times (illustrated in Green, Orange, and Blue in Figure 6G). These cell assemblies were embedded in the background Poisson firing at the rate of 1 Hz in 60 s. We have clustered the data with different alpha and same clustering parameter. The result indicates that our method with α = 0.1 detects both types of cell assemblies as identical when shrinkage factor is three and five. We used MinPts 5 and v 5 for the clustering.

### 3.3. Place-Cell Firing Sequences in the Hippocampus

**Figure 7:**
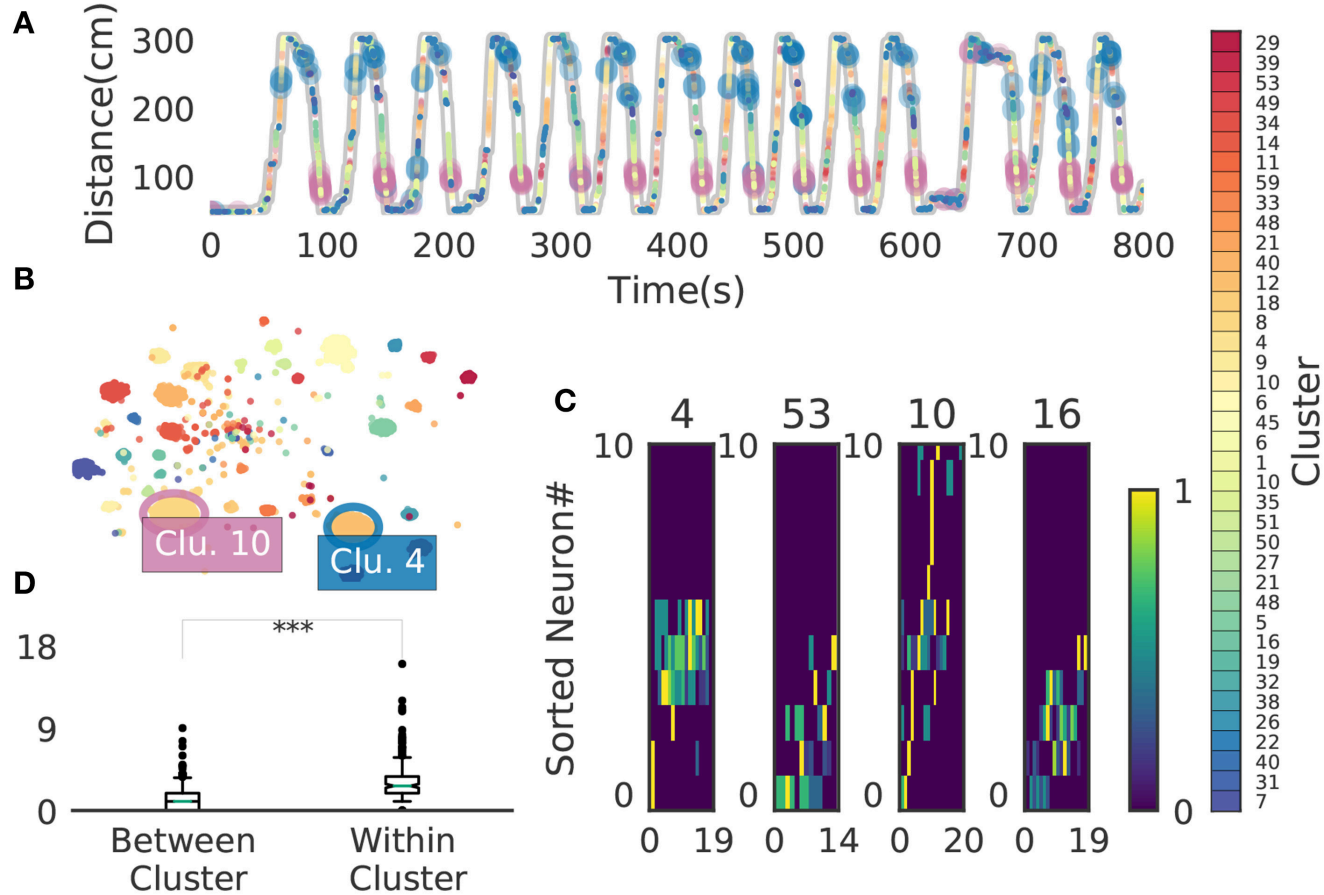
Cell assemblies extracted from hippocampal CA1. **(A)** The spatial locations in the linear maze are shown for the detected cell-assembly sequences. The x-axis shows time and y-axis shows the position of the rat. Twenty Go clusters and 20 Back clusters of time windows are shown, and the spatial positions at which they were detected were indicated by the rightmost color diagram. Each colored region shows where each segment was observed. Cluster four and ten were additionally colored in blue and pink. Cluster indices are shown on the right, which were sorted and colored according the order of appearance during the going and returning along the maze. We sorted cluster indices according to their mean kernel density estimate (which is shown in Figure 9). The window size was 100 ms. **(B)** t-SNE visualization of the feature space. Note that most of the neighboring colors in A are also adjacent in B, suggesting that two neuronal activities observed at contiguous spatial positions have similar temporal patterns, but are still separate enough in the feature space. **(C)** Profiles of cell-assembly sequences are shown for four cluster (left four panels). The first 10 neurons for four profiles are shown with number indicating cluster identity (Profile 4: [41, 0, 85, 53, 59, 68, 76, 5, 54, 4], Profile 53: [4, 15, 68, 49, 26, 84, 77, 12, 82, 13], Profile 10: [26, 54, 84, 17, 76, 68, 60, 36, 15, 0], Profile 16: [82, 39, 83, 61, 48, 13, 77, 28, 26, 85]). Color indicates the firing rate of each neuron after a normalization across neurons within the profile: from lowest (dark blue) to highest (yellow). The cells (y-axis) were sorted according to the relative temporal order note that the first ten neurons represent different firing order in the different profiles. (x-axis) of the peak activity of each cell in each profile. Note that the absolute length of the x-axis in each profile does not necessarily represent the actual temporal length of sequences, though the approximate length coincides the width of temporal windows (100ms in this case). **(D)** Between-cluster and within-cluster edit similarity scores. Edit similarity was compared between time windows belonging to the same cluster and those belonging to different clusters. Box plot with whiskers from 5 percentile to 95 percentiles are shown with outliers (filled circles).

We now demonstrate that our method enables an automatic detection of firing sequences of rat hippocampal neurons during spatial exploration (Mizuseki et al., 2009). Our method extracted 60 distinct clusters of data segments (i.e., time windows), each of which appeared repeatedly at a different location in the maze and in a specific movement direction (Figure 7A). We introduced three labels (Go, Back, Stop) for categorizing these clusters in terms of their dominant relationships to behavior, allowing each cluster to have multiple behavioral labels (section 2). Twenty clusters appeared primarily when the rat ran from the start to the goal (we call it the Go cluster), 20 clusters appeared primarily during the opposite movement (called the Back cluster). There are five overlapping clusters between the two types (#6, #10, #21, #40, #48). Another 20 clusters mainly appeared during immobility (Stop cluster). The relationship between each cluster and a behavioral state indicates that our method successfully detected behaviorally relevant cell assemblies, which likely consist of hippocampal place cells. Some clusters (e.g., clusters #4 and #10) were detected during both locomotion and the resting state. These patterns presumably correspond to place-cell firing phase-locked to theta oscillation and their ripple-associated replays, respectively (Nádasdy et al., 1999; Foster and Wilson, 2006). It is notable that our method automatically extracted these sequences in spite of the different time scale. Such reactivation was also observed in (Mizuseki et al., 2009). Figure 7B shows visualization of the feature space with t-SNE, which defines a mapping from high-dimensional data space to a low-dimensional space for visualization such that the spatial relationships between data points are optimally preserved (Maaten and Hinton, 2008). We constructed the core temporal structure, which termed profile, of highly variable activity patterns of each cell assembly (section 2). Figure 7C displays four sample profiles of activity patterns for the clusters detected, where only the first 10 neurons are shown. Three examples (#4, #16, #53) show clusters each of which was observed just once along the maze (see the rightmost color diagram) whereas cluster #10 appeared at two slightly different places during movements in both directions.

**Figure 8:**
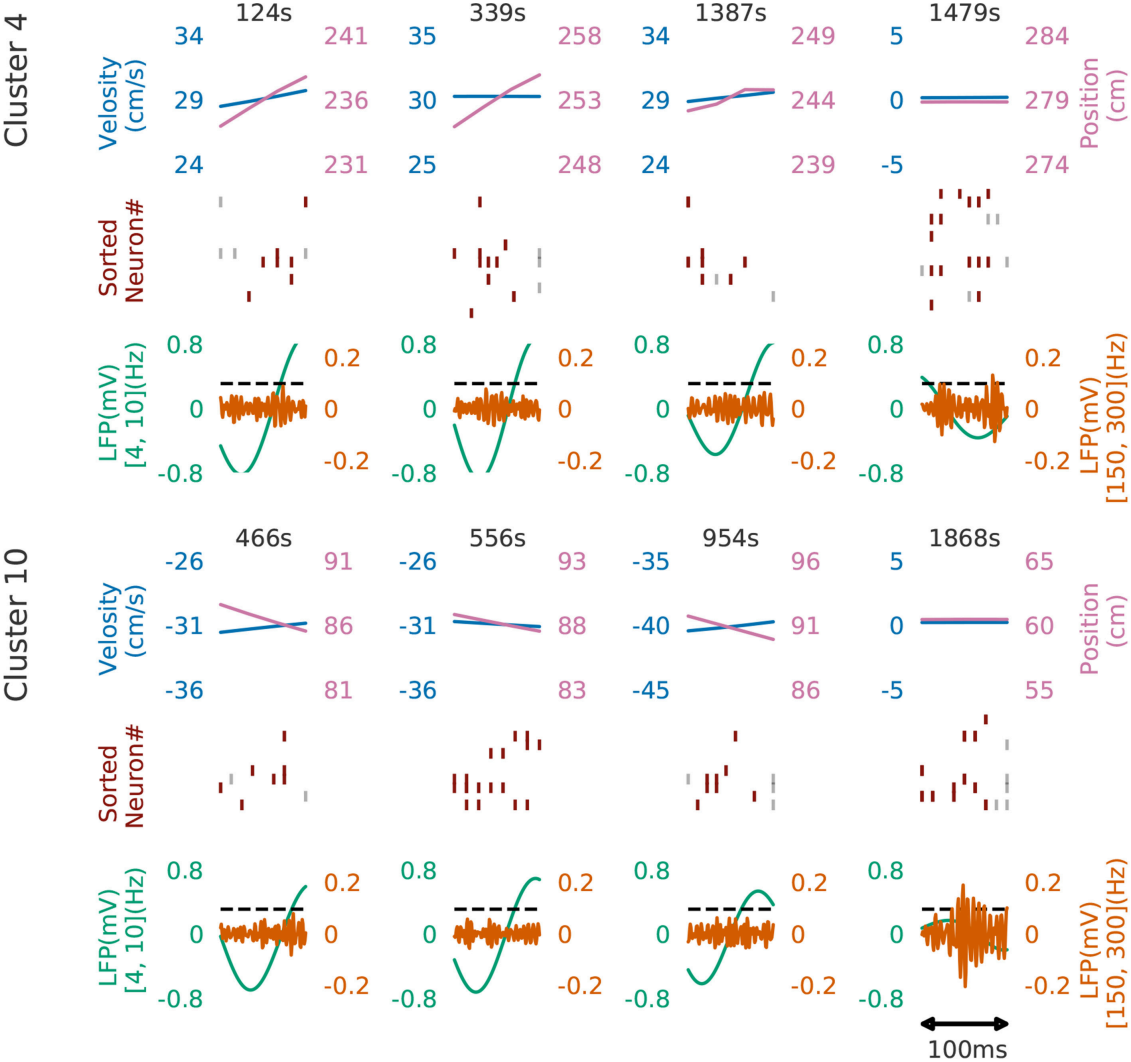
Cell assemblies detected from CA1. Four examples are shown from cluster 4 (top) and cluster 10 (bottom). The top, middle and bottom panels display the velocity and spatial position of the rat, spike raster, and local field potentials band-passed at 4–10 Hz (theta band) and 150–200 Hz (sharp-wave ripples). We calculated criteria (0.095) of the ripple detection with the method described in Davidson et al. (2009) to confirm our detected patterns during immobility accompanied with sharp wave ripple. In the middle panel, gray vertical bars show noisy spikes and red bars represent the core spikes of the corresponding profile. Neurons are sorted according to their firing position within the average profile.

Figure 8 shows four examples of spike rasters from the extracted cell assemblies corresponding to two clusters (cluster 4 and cluster 10) together with the position and velocity of the animal. In each cluster, the spatiotemporal activity patterns vary from segment to segment, but they also resemble each other (see Figure 7D for the statistics of within-cluster and between-cluster similarity of activity patterns). Thus, our method is robust against changes in the temporal scale of sequences. In addition, each of the two clusters include an example of replays (at 1479s in cluster 4 and at 1868s in cluster 10) of a cell assembly in the immobile state of the animal. For the parameter values used here, these sequences were grouped into the same cluster because our method allows a certain degree of spike timing jitters. The larger the value of α, the stricter the penalty for spike timing jitters. We quantitatively evaluated clustering performance at different values of α to find that both the number of clusters (*N*_*c*_) and the total number of time windows (*N*_*tw*_) in the clusters decreased as the value of α was increased: at α = 0.1, *N*_*c*_ = 58 and *N*_*tw*_ = 21162; at α = 1.0, *N*_*cl*_ = 46 and *N*_*tw*_ = 17858; at α = 10.0, *N*_*c*_ = 33 and *N*_*tw*_ = 8028.

**Figure 9:**
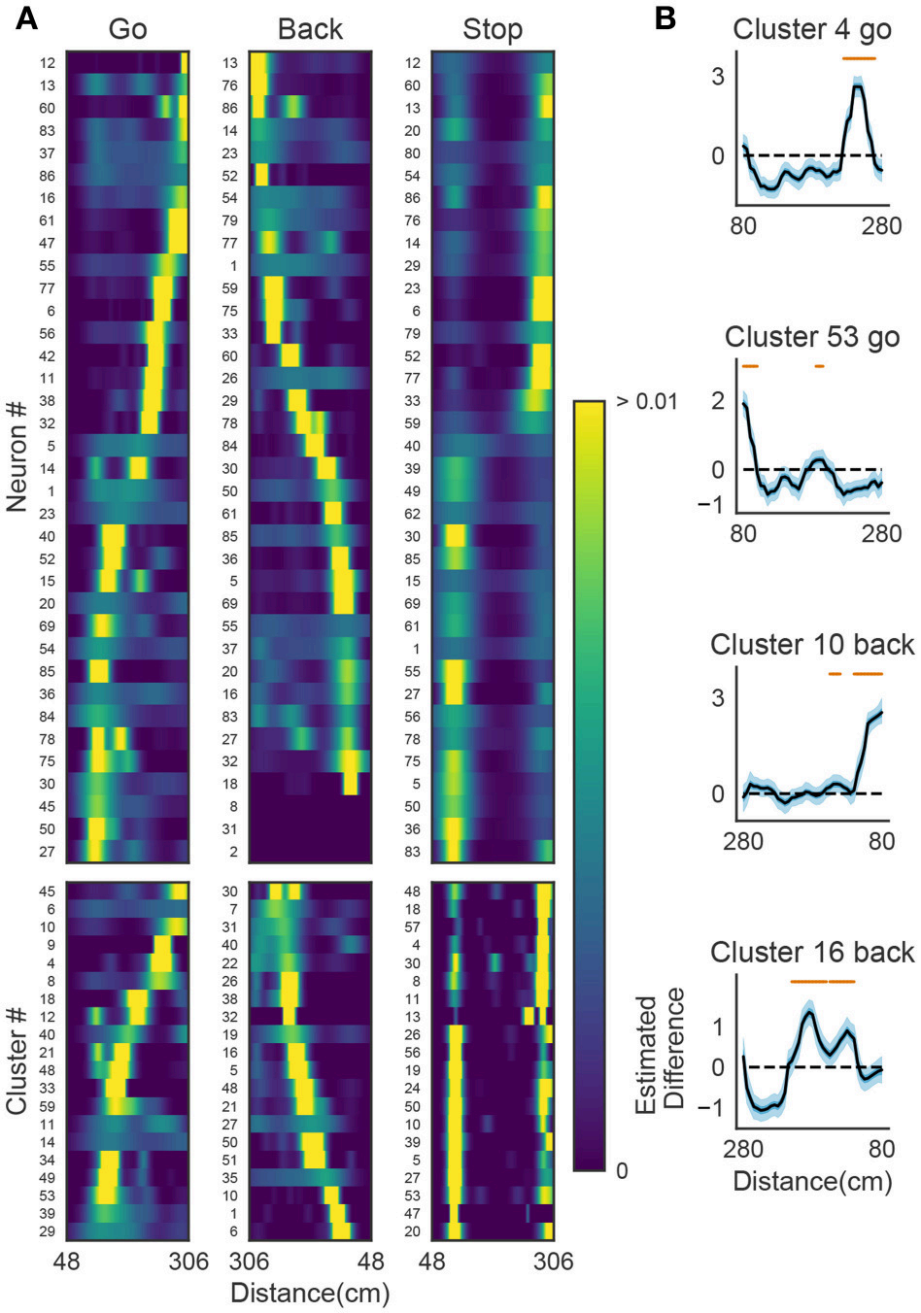
The relationships between hippocampal cell-assembly sequences. **(A)** The spatial receptive fields are shown for 36 hippocampal neurons (top) and cell assemblies belonging to the 20 clusters (bottom). The pseudo-color code indicates the probability of firing. **(B)** Estimated edit similarity difference between the original and shuffled data. Each similarity scores were calculated by using the profiles of cell assemblies and spike trains of hippocampal neural population using a sliding time window. Solid lines correspond to the mean of the estimated differences and blue shaded regions to 95% credible interval. Orange bars designate the spatial locations at which the lower bound of the credible interval is positive.

Figure 9A shows the receptive fields of neurons (top panels) and clusters (bottom panels) when the rat was running forward, backward and stopping. It is suggested that a cluster detected at a given spatial location consists of neurons having similar receptive fields around the location. To examine whether the detected sequences have significant relationships to behavior, we generated shuffled neural data in which spikes of each neuron were redistributed at randomly chosen temporal locations according to a homogeneous Poisson process. This manipulation preserved the average firing rates of individual neurons. Edit similarity score for cell-assembly sequences at some locations was significantly higher than the score calculated from the shuffled data (Figure 9B) (see Bayesian modeling section in section 2). The result suggests that the cell-assembly sequences and their profiles actually captured the behaviorally relevant characteristics of neural population activity. Section 2 explains the details of the statistical model used for the analysis.

### 3.4. Cell Assemblies in the Prefrontal Cortex

**Figure 10:**
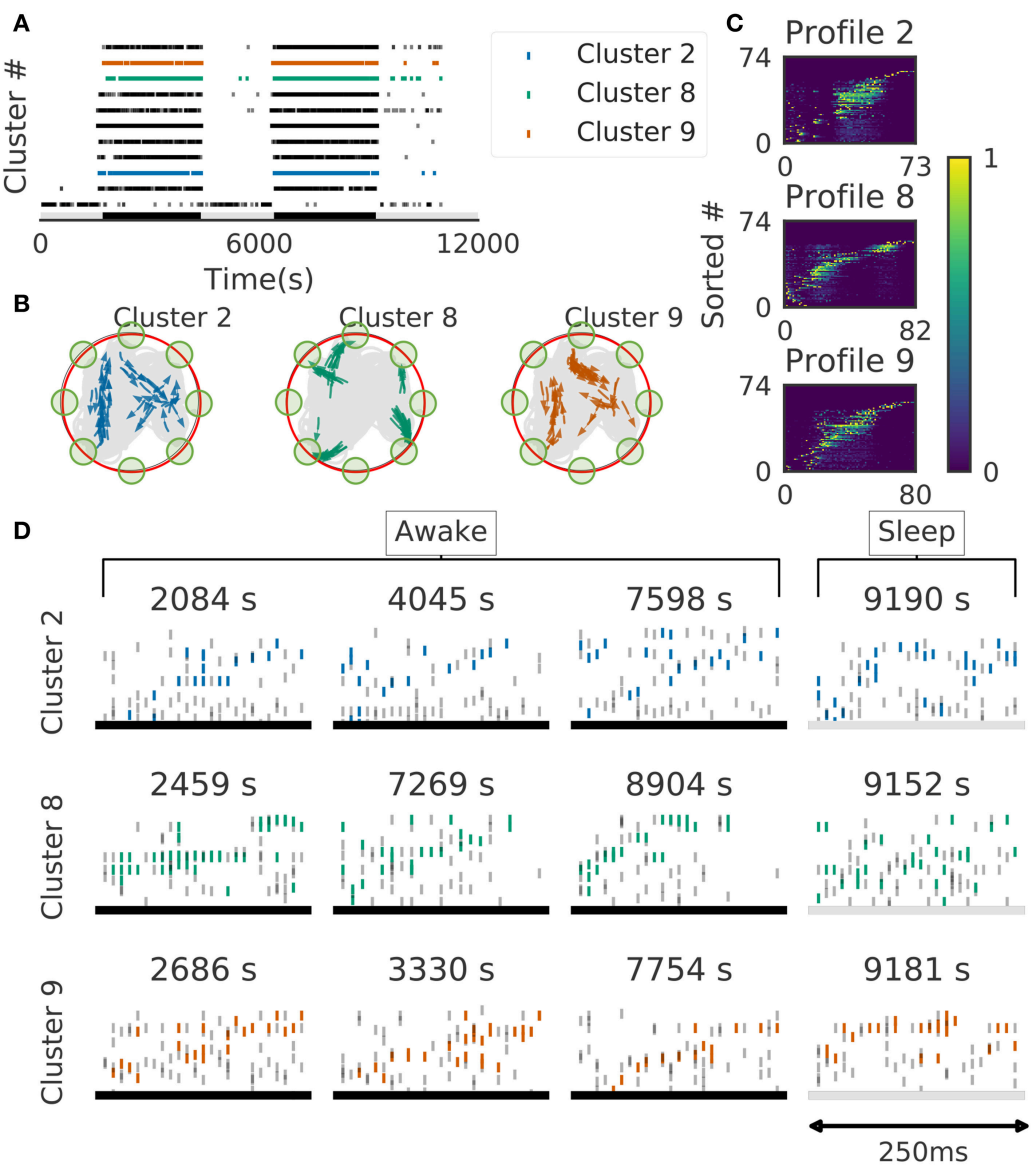
Cell assemblies detected from the prefrontal cortex. The width of time windows was 250 ms. **(A)** The onset times of detected time windows are shown for all the clusters. **(B)** The spatial positions and movement directions of a rat are shown at the onset times of detected time windows belonging to three clusters by arrows. Only ten percent of randomly sampled elements are drawn for visualization. **(C)** Profiles are shown for three prefrontal cell assemblies in terms of the sorted neuron id and relative temporal order. The approximate length of the x-axis coincides the width of temporal windows (250 ms). **(D)** Cell assembly sequences detected in awake (left three panels) and sleep (rightmost panel) are shown for the three clusters. From top to bottom, each row corresponds to the profile 2, 8, and 9, respectively. Some sleep replay events showed evidence of multiple replays within the 250 ms window. This is most apparent in the first row, where the upward ramp is seen twice.

We further validated the method in neural ensemble activity recorded from the medial prefrontal cortex of rats performing a memory-guided spatial sequence task (Euston et al., 2007): see the paper for experimental details). Briefly, the rats were trained to visit eight locations equally spaced around the perimeter of a circular arena in a prescribed sequential order with electrical brain stimulation as a reward. Our method detected 11 clusters of prefrontal cell-assembly sequences in total (Figure 10A). The previous analysis based on template matching revealed a sequence and its replay pattern in the same rat as we analyzed here (Euston et al., 2007). Though some of the detected clusters are overlapped, the larger number of detected clusters indicate that the method extracted activity patterns without any reference to events or positions on the track. These clusters were detected in both behaving state and sleep state, and some clusters were frequently replayed during sleep (Figure 10A). During the behavior, cell assemblies were typically found when the rats were approaching or leaving a reward zone (Figure 10B, green circles). The profiles of three cell assemblies are shown in Figure 10C. Each sequence usually appeared just once in a 250ms window during behaving state, whereas they were repeated multiple times during sleep state (Figure 10D). Thus, the detected sequences were time compressed during sleep. These results are consistent with the previous findings (Euston et al., 2007).

**Figure 11:**
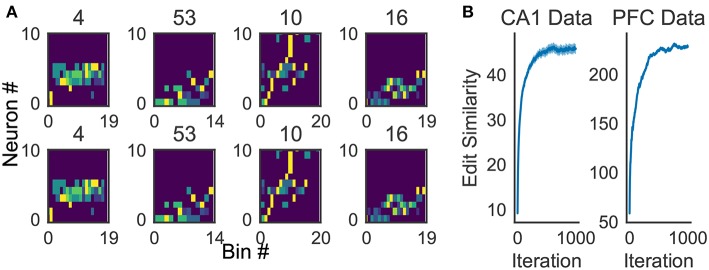
Characteristics of the proposed algorithm **(A)** There is no visual difference between the implementation used in our framework and described in Barton and Sternberg (1987) (bottom). **(B)** Convergence of profile evaluation. Profiles were calculated for all clusters in the activity data from CA1 and PFC. The abscissa shows the number of iterations and the ordinate shows edit similarity between the current profile and the preceding one. Thick lines represent the means over all clusters and shaded areas show the standard errors.

Because the profiling method is essential for inspecting the activity patterns of cell assemblies, we examined whether the method works robustly. For the applications reported in this study, 10*x* to 100*x* times updating, where *x* is the number of members of the cluster to be profiled, maximized the average edit similarity score between the individual cluster members and the corresponding profile. The convergence of the profiling procedure is shown in Figure 11A for experimental data.

### 3.5. Comparison With a Recent Method for Sequence Detection

Recently, a statistical method to extract assembly structure with arbitrary constellations of time lags was proposed (Russo et al., 2017). We compared our method with the method (the code is available at https://github.com/DurstewitzLab/Cell-Assembly-Detection). The method recursively combines neurons into larger sets based on significant statistical relations between their activities. We first compared performance on artificial spike data in which a spike sequence of 10 neurons was embedded into background Poisson spike trains of total 100 neurons (including the ten). The target sequence pattern occurred 60 times and background firing rate were 1 Hz. We generated two sets of 50 independent datasets: one with the sequence duration of 100 ms and the other with 500 ms. The length of each dataset was 60 sec.

**Figure 12:**
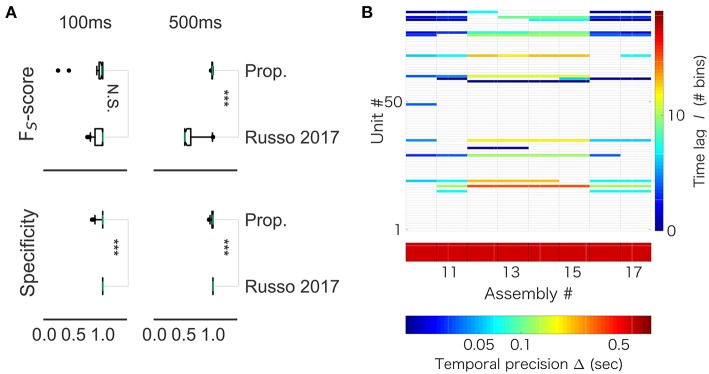
Comparison with Russo 2017 **(A)** *F*_S_-score (top) was significantly higher in the proposed method than in Russo et al. (2017) for artificial data of length 500 ms (right, *p*-value is 1.831e-12), but not significantly different between the two methods for artificial data of length 100 ms (left, *p*-value is 0.6386). **(B)** The output of Russo 2017 is shown. The distributed computer code of Russo 2017 analyzes spike train data at various temporal precisions (i.e., correlation time scales) ranging from milliseconds to several seconds. Only the part of results is shown to clarify the characteristic property of Russo 2017: the eight cell assemblies classified as number 10 to number 17 look very similar to each other.

While our method robustly showed a near perfect detection, performance of the previous method depended on particular samples. For 100 ms-long sequences, the mean *F*_US_ value was 0.940 and the variance was 0.134 in our method, whereas the mean *F*_US_ value was 0.940 and the variance was 0.090 in the method by (Russo et al., 2017). For 500 ms-long sequences, while our method yielded the mean *F*_US_ value of 0.989 and the variance of 0.008, the method by (Russo et al., 2017) showed the mean *F*_US_ value of 0.635 and the variance of 0.197. Thus, the two methods worked equally well for the shorter sequences (Figure 12A, left), but our method exhibited better scores than the previous method for the longer sequences (Figure 12A, right). On the other hand, the previous method yielded better specificity than our method for both data lengths (left, *p* = 0.001174; right, *p* = 4.353e-07). This result suggests that the previous method is more conservative, and it produces less false positive. However, the differences were subtle: 99% confidence interval is (-0.0333, 0.0000) and (-0.0166, 0.0000) for the lengths of 100 and 500 ms, respectively. In addition, we analyzed spike data obtained in the rat hippocampus (CRCNS.org., http://dx.doi.org/10.6080/K09G5JRZ), which highlighted another difference between the two methods. As shown in Figure 12B, our method detected multiple cell assemblies that cover the entire linear track. In contrast, their method failed to merge similar subsequences into a small number of core sequences. For instance, see assemblies #12 to #15, which consisted of similar neuronal populations with similar time lags. The same can be said for assemblies #10, #11 and #16. This excessive division is presumably due to jitters and failures in spike generation. Although pruning solutions called “biggest” and “distance” were described in (Russo et al., 2017), the previous method was not completely free from the difficulty at least in our analysis of real data.

### 3.6. Computational Time

This section lists up the computational time needed to detect cell assembly sequences from each dataset. All computations were done on Mac Pro (Late 2013) with 2.7 GHz 12-Core Intel Xeon E5 and 64GB RAM.

Our method took 18 h to process the data described in 3.3 (Figures 7, 8), whereas the method described in Russo et al. (2017) took 28 h (result is shown in Figure 12).

To analyze 300 s-length artificial data, our method took 27 min std. 95 s while the PCA/ICA-based method took 1 min std. 2 s (Figures 6A–C). On 60 s-length artificial data, our method took 5 min std. 63 s while the PCA/ICA-based method took 47 s std. 1 s (Figure 6E).

Our method took 8 h to process the data described in section 3.4 (Figure 10).

## 4. Discussion

In this study, we have developed a method for extracting multiple repeated sequences of cell assemblies from multi-neuron activity data. Our method is based on edit similarity, which was developed in computer science as a measure of similarity between strings. Edit similarity compares the serial order of common elements appearing in two strings with or without discounting variations in inter-element intervals, hence it provides a flexible and efficient metric for comparing highly noisy spatiotemporal activity patterns of cell assemblies. We have validated the method first in artificial data and then in neural activity data recorded from the hippocampus and the prefrontal cortex of behaving rodents.

In the assessment with artificial data, we showed that our method is superior to PCA- (Peyrache et al., 2009; Lopes-dos Santos et al., 2011) and ICA-based methods (Laubach et al., 1999; Lopes-dos Santos et al., 2013) in detecting an assembly of synchronously firing cells when the cell-assembly structure is clear (i.e., small timing jitters of ±10 ms). However, when timing jitters are fairly large (i.e., ±50 ms), our method tends to categorize such a cell assembly into multiple clusters and the performance becomes inferior to PCA/ICA-based methods. However, this does not show the weakness of the method because it is constructed as such: the method is specialized for sequence detection. Dynamic programming-based methods were previously introduced to quantify the similarity between spike trains of neurons(Victor and Purpura, 1996; Victor et al., 2007). To our knowledge, no methods have been developed with edit similarity to detect similar sequences of cell assemblies from noisy population neural data in unsupervised manner. Other methods also exist and discovered the activation of specific neuron ensembles (Lee and Wilson, 2002; Ohki et al., 2005; Chen and Wilson, 2017). The previous methods, however, are generally not effective when data has a low signal-to-noise ratio, for instance, when most of the recorded neurons do not participate in sequences. In addition, the previous methods have difficulties in distinguishing partially overlapping cell-assemblies.

In particular, our method enables blind detection of cell-assembly sequences without referring to external events such as sensory stimuli and behavioral responses. Recently, a novel statistical approach was proposed for the detection of cell assembly structure with multiple time scales (Russo et al., 2017). Starting from pairwise correlations in neuron pairs, the method finds significantly correlated neurons within the set of cell assemblies detected at the previous step. Acceleration of the analysis was achieved by discarding statistically less significant combinations at the next step. However, in the successive statistical tests, the detection of long sequences becomes rare and time consuming. In contrast, our method is computationally more efficient when searching longer cell-assembly sequences. It may also be inappropriate to discard long sequences just because they are statistically less significant. For instance, place-cell sequences spanning several seconds of behavior emerge in the hippocampus during spontaneous activity after spatial experience (Dragoi and Tonegawa, 2013; Grosmark and Buzsáki, 2016). We propose that behaviorally relevant cell-assembly sequences should be addressed after all possible candidates have been identified. Our method enables such an analysis of cell-assembly sequences.

We note that the two data examples analyzed here (Euston et al., 2007; Mizuseki et al., 2009) were recorded during stereotyped, repeated behaviors, which presumably entrained similar repeated patterns in neural activity. Whether the present algorithm can be used to detect spontaneous (as opposed to stimulus- or activity-driven) patterns, such as those reported by Luczak et al. (2007) and Luczak et al. (2009) remains to be tested. Other intriguing extensions of this method include the detection of hierarchically organized cell assemblies over multiple spatiotemporal scales. Such an extension requires a flexible on-line tuning of time windows, which is a challenge at the moment. One area where time-scaling would be particularly relevant is in the detection of replay events, which often occur at a compressed timescale during slow-wave sleep. Our method might detect considerably more reactivation events if we adjust the temporal scaling between behavior and sleep epochs. We also note that in principle our method is applicable to optical imaging data if we adequately tune the sizes of time window and temporal discount factor.

In summary, we proposed a novel method for the blind detection of cell-assembly sequences based on the edit similarity score and an exponential discount for timing jitters. This method does not rely on external references, and is therefore useful for detecting not only externally driven firing sequences, but also internally driven sequences emergent from arbitrary mental procedures. Whether the method reveals the involvement of cell-assembly sequences in mental processes is an interesting open question.

## Author Contributions

TF supervised the work. TF and KW conceptualized the work. KW, TH, and TF developed the mathematical method. The comparison between the proposed method and Russo 2017 was done by KW and MT. Other data analysis was done by KW and MT, and DE provided the data from the prefrontal cortex and contributed to the calibration of the method. TF and KW wrote the initial draft and all authors edited the manuscript.

### Conflict of Interest Statement

The authors declare that the research was conducted in the absence of any commercial or financial relationships that could be construed as a potential conflict of interest.
